# Ugly ducklings—the dark side of plastic materials in contact with potable water

**DOI:** 10.1038/s41522-018-0050-9

**Published:** 2018-03-27

**Authors:** Lisa Neu, Carola Bänziger, Caitlin R. Proctor, Ya Zhang, Wen-Tso Liu, Frederik Hammes

**Affiliations:** 1 Eawag, Swiss Federal Institute of Aquatic Science and Technology, Dübendorf, Switzerland; 2 Department of Environmental Systems Science, Institute of Biogeochemistry and Pollutant Dynamics, ETH Zürich, Zürich, Switzerland; 3 Department of Civil and Environmental Engineering, University of Illinois at Urbana-Champaign, Urbana-Champaign, USA

## Abstract

Bath toys pose an interesting link between flexible plastic materials, potable water, external microbial and nutrient contamination, and potentially vulnerable end-users. Here, we characterized biofilm communities inside 19 bath toys used under real conditions. In addition, some determinants for biofilm formation were assessed, using six identical bath toys under controlled conditions with either clean water prior to bathing or dirty water after bathing. All examined bath toys revealed notable biofilms on their inner surface, with average total bacterial numbers of 5.5 × 10^6^ cells/cm^2^ (clean water controls), 9.5 × 10^6^ cells/cm^2^ (real bath toys), and 7.3 × 10^7^ cells/cm^2^ (dirty water controls). Bacterial community compositions were diverse, showing many rare taxa in real bath toys and rather distinct communities in control bath toys, with a noticeable difference between clean and dirty water control biofilms. Fungi were identified in 58% of all real bath toys and in all dirty water control toys. Based on the comparison of clean water and dirty water control bath toys, we argue that bath toy biofilms are influenced by (1) the organic carbon leaching from the flexible plastic material, (2) the chemical and biological tap water quality, (3) additional nutrients from care products and human body fluids in the bath water, as well as, (4) additional bacteria from dirt and/or the end-users’ microbiome. The present study gives a detailed characterization of bath toy biofilms and a better understanding of determinants for biofilm formation and development in systems comprising plastic materials in contact with potable water.

## Microbiome: bath toys support microbial growth

While bathing typically means good hygiene, bath toys can serve as incubators for microbial growth. Microbes colonize nearly every natural and human-made surface, sometimes living within complex communities called biofilms. A team led by Frederik Hammes at the Swiss Federal Institute of Aquatic Science and Technology found that tap water bacteria and fungi readily formed biofilms inside bath toys, suggesting that bathing provides food for microbes. These nutrients may come from bath toys’ polymeric material, from care products like soap and from human secretions like sweat. While 16S rRNA sequence analysis found that some of the microbes were related to disease-causing strains, future work is needed to assess the disease risk from these bath toy-associated biofilms. This work sheds light on how microbes are spread by our routine activities and that we are bathed in microbes, literally.

## Introduction

Unwanted microbial growth in the built environment is frequently reported. Bathroom conditions in particular are known to promote biofilm formation and growth due to moderately high temperatures and increased humidity.^1,2^ In this regard, unwanted microbial growth has been reported, e.g., for basins, bath tubs, and drains,^3,4^ as well as for shower fixtures^5–7^ and shower curtains.^8^ In the same environment, bath toys, best known for so-called “rubber ducks”, present an interesting junction between potentially vulnerable end-users and several determining factors for such growth, namely (1) low-quality polymeric material, (2) potable water from the building plumbing, and (3) additional nutrients and microbial contamination by bathing.

Synthetic polymeric materials in contact with potable water not only adsorb some organic matter from the water,^9^ but also release substantial amounts of organic carbon through migration, leakage, leaching, and/or permeation, including, e.g., plasticizers, stabilizers and antioxidants.^10–13^ A fraction of this organic carbon is biodegradable and offers microorganisms a significant source of assimilable organic carbon (AOC).^14–16^ This AOC in turn promotes microbial growth and biofilm formation^16–19^ and influences the microbial community composition.^5,20,21^ Flexible polymeric (i.e., plastic) materials, which are typically used in the production of bath toys, are particularly known for excessive carbon leaching and unwanted biofilm formation and growth.^16,20^

One source of pioneer microorganisms for bath toy biofilms is the tap water microbiome, which differs substantially between different locations.^22,23^ Tap water comprises complex microbial communities and in many cases also opportunistic pathogens, such as *Pseudomonas aeruginosa*,^24–26^ *Legionella pneumophila*,^27^ and *Mycobacterium avium*.^28^ However, nutrients from tap water typically do not contribute to excessive microbial growth, as it is an oligotrophic environment.^29,30^

A second, and potentially more dominant, source of microorganisms is the used bath water, which exposes bath toys to microorganisms from both the human microbiome as well as from external/environmental microbial contamination.^22,31^ In addition, bath water is a substantial source of supplementary organic and inorganic nutrients, introduced by care products (soap, shampoo, conditioner) and the human body itself, e.g., in form of urine residuals.^32,33^

Apart from esthetic issues, potential problems with contaminated bath toys have been recognized before. Several decades ago, a study by Ruschke^2^ suggested that bath toys not only facilitate microbial growth, but specifically the proliferation of opportunistic pathogens and unwanted organisms, such as *P. aeruginosa* or *Enterococcus* spp. Approximately 20 years later, a multidrug-resistant *P. aeruginosa* outbreak in a children’s hospital was linked to shared bath toys.^34^ That study showed that *Pseudomonas* spp. was only present in the bath toys and not detectable in the bath water itself, making this the first connection between plastic bath toys and children’s infections. While scientific studies on the topic are limited, many parents are seemingly well aware of this biofouling phenomenon, which is evidenced by numerous internet blogs and discussion groups on the topic (e.g., www.blogs.babycenter.com or www.welovebeingmoms.blogspot.ch; Table S1).

The aims of the present study were (1) to provide a comprehensive characterization of biofilms grown on the inside of real bath toys to elucidate this phenomenon, and (2) to establish a better understanding of the factors that drive the development of these biofilms. For the first, we studied biofilms from used bath toys that were collected from random households (real bath toys). These were characterized by their appearance, microbial abundance and community composition. For the second, we examined and compared biofilms that were established in new, identical bath toys under controlled conditions simulating actual use in clean and dirty bath water (control bath toys). The resulting data enabled conclusions on the impact of material composition, water characteristics, and external contamination on biofilm formation in these unique environments, as well as recommendations to mitigate the potential microbial risks for vulnerable users.

## Results

### Visible and dense biofilms inside all bath toys

**Fig. 1:**
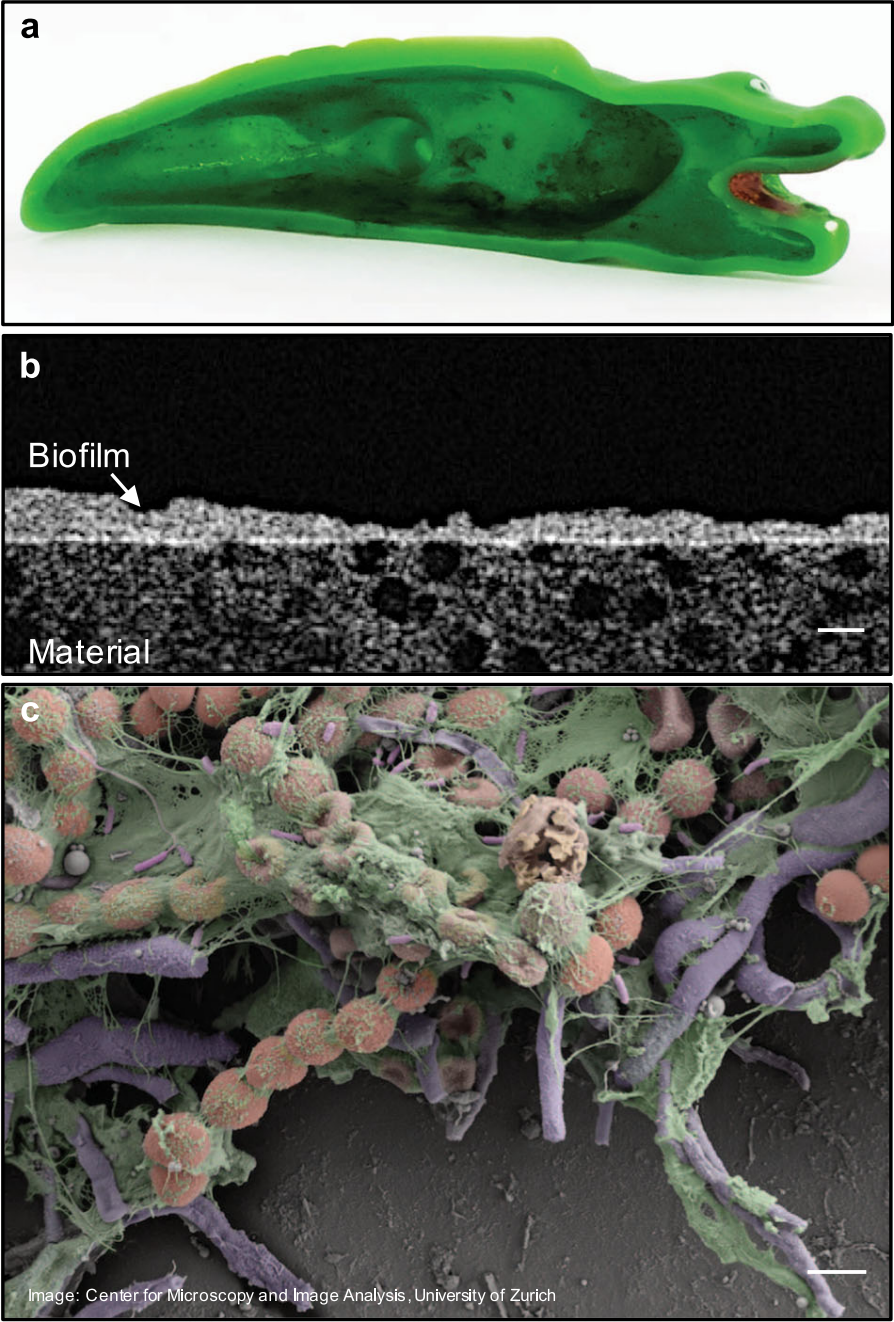
Visualization of biofilms on the inner surface of bath toys. **a** The inner surface of a bath toy used under real conditions. **b** Optical coherence tomography image of the biofilm structure and thickness of the same bath toy (scale bar: 50 µm). **c** Scanning electron microscopy image revealing the complex structure and composition of these bath toy biofilms. Colors were added artificially to draw attention to varied structures (scale bar: 2 µm). For additional images, see supplementary information

All bath toys analyzed in this study had dense and slimy biofilms on the inner surface (Fig. 1a, Figure S1 for images of all bath toys). While most of the real bath toy biofilms (~70%) had areas of black discoloration (indicative of mold growth), biofilms inside the control bath toys were transparent. The visual biofilm observation was confirmed by optical coherence tomography (OCT) analysis on selected bath toys, revealing heterogeneous biofilm shapes and thicknesses both within and between individual toys, ranging up to 100 µm (Fig. 1b, Figure S2 for additional images). High resolution scanning electron microscopy (SEM) imaging of selected toys showed complex biofilm compositions with what appeared to be diverse microorganisms in a thick layer of extracellular polymeric substances (Fig. 1c, Figure S3 for additional images).

### High numbers of bacteria in bath toy biofilms

**Fig. 2:**
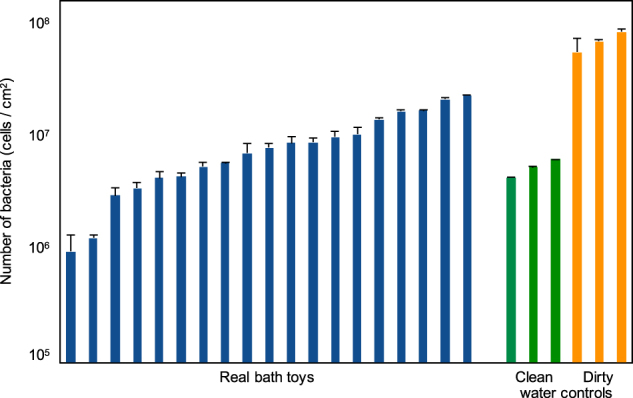
Number of bacteria in biofilms from the inner surface of bath toys. Flow cytometry was used to determine the number of bacterial cells in bath toy biofilms using SYBR^®^ Green I staining following biofilm removal and dispersal. Bath toys were either from real households (real bath toys), or used under controlled conditions with clean water prior to bathing (clean water controls) or with used water after bathing (dirty water controls). Error bars represent standard deviation of triplicate measurements

All bath toy biofilms showed high but variable numbers of bacteria. The real bath toy biofilms had an average coverage of 9.5 × 10^6^ cells/cm^2^ (range: 0.1–2 × 10^7^ cells/cm^2^), which equals an average of 1.3 ± 0.07 × 10^9^ cells/bath toy (*n* = 19) when calculated with the surface area of individual toys (Fig. 2, Figure S4). In comparison, clean water controls were on average covered with approximately half that number of cells (5.5 ± 0.08 × 10^6^ cells/cm^2^; *n* = 3), which equals 1.2 ± 0.03 × 10^9^ cells/bath toy (*n* = 3). The highest coverage was observed on dirty water control toys with an average of 7.3 ± 1.0 × 10^7^ cells/cm^2^ (*n* = 3), or 1.3 ± 0.2 × 10^10^ cells/bath toy (*n* = 3), respectively. Cell numbers in the dirty water controls were shown to be tenfold higher in magnitude than in the clean water controls (ANOVA, *F*-test, *p*-value 0.009; Shapiro–Wilk normality test). Viability analysis showed that the percentage of intact cells was on average 62.8 ± 19.2% (*n* = 19) in real bath toys, 27.2 ± 14.2% (*n* = 3) in clean, and 20.3 ± 5.7% (*n* = 3) in dirty water controls (Figure S4). The higher average value for intact cells in real bath toy biofilms was not statistically significant, neither against clean (ANOVA, *F*-Test, *p* = 0.82) nor against dirty water controls (ANOVA, *F*-Test, *p* = 0.17).

### Diverse microbial communities in bath toy biofilms

#### Similarities and differences between biofilm bacterial communities

Overall, bath toy biofilms showed diverse communities. Remarkably, only eight out of a total of 12,229 operational taxonomic units (OTUs) were shared between all bath toys (i.e., control and real bath toys) (Table S2). Four of these OTUs could be classified on genus level, namely *Bradyrhizobium* spp., *Agrobacterium* spp., *Caulobacter* spp., and *Sphingomonas* spp. The remaining OTUs belonged to the families Methylobacteriaceae, Comamonadaceae, and Microbacteriaceae, but could not be further identified to genus level (for more information see Table S2).

**Fig. 3:**
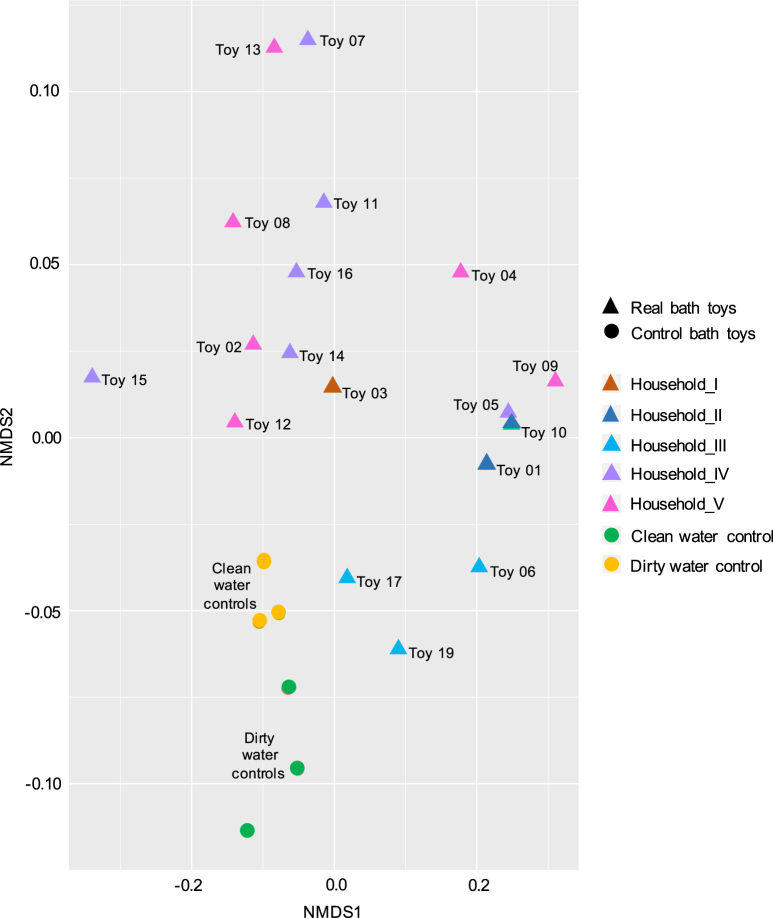
Non-metric multidimensional scaling (NMDS) plot to assess similarities in bacterial community compositions between bath toy biofilms. Filtered OTU sequences, scaled to an even sampling depth, were ordinated with the NMDS method using the Bray–Curtis distance matrix. Triangles represent real bath toy and circles control bath toy biofilm communities. Color codes for real bath toy samples indicate origination from five different Swiss households, while control bath toys are separated into clean and dirty water controls

For the comparison of bacterial communities between single bath toys, a non-metric multidimensional scaling plot was used (Fig. 3). Distances in this plot correlate with the degree of similarities between the biofilm communities, based on the similarity and frequency of OTUs detected in each of them. Samples that cluster closer to each other have a higher degree of similarity (e.g., Toy05 and Toy10) than samples that cluster further apart (e.g., Toy13 and Toy19; Fig. 3).

Real bath toys showed a diverse clustering, clearly indicating a different community composition in each toy (Fig. 3). Yet, biofilm communities from multiple bath toys originating from one household showed variable levels of similarity. For example, the three samples from “Household_III” clustered closer to each other than the six from “Household_V”, indicating more similarities within samples from “Household_III”. Shared OTUs within bath toys from the same household varied between 1445 (“Household_II”, *n* = 2) and 29 (“Household_VI”, *n* = 6) (Table S3). Moreover, household-specific core communities could be identified (i.e., OTUs which were found in bath toy biofilms from a single household, but which were not found in other households; Table S3). However, this “household specific core” represented only about 2.1 ± 1.4% (*n* = 5) of the total number of reads in any given house. This result is inconclusive as the analysis is limited by the low number of samples in each household. Overall, only 13 OTUs were shared between real bath toy biofilms. These included the OTUs shared by all bath toy samples (see above), as well as *Methylobacterium* spp. and *Novosphingobium* spp., and the family Hyphomonadaceae (Table S2).

For the control bath toy biofilms, samples from clean water controls clustered separately from dirty water controls (Fig. 3), with smaller distances between clean water control replicates compared to those of the dirty water controls. Forty-seven OTUs were shared amongst all control bath toys (Table S2). However, this number increases when distinguishing between clean and dirty water controls, with 72 shared OTUs in clean and 107 in dirty water controls. Most of the shared OTUs were representatives of the families that were already identified for real bath toys (see above). Additional shared OTUs belonged to diverse taxa within families such as Rhodobacterteraceae, Rhodospirillaceae, Pseudomonadaceae, Bdellovibrionaceae, and Flavobacteriaceae. The shared OTUs diverged on closer classification, with, e.g., the genera *Methylobacterium* spp., *Streptococcus* spp., and *Planctomycetes* spp. well represented in clean water controls, and the genera *Rhodobacter* spp., *Mycobacterium* spp., and *Delftia* spp. well represented in dirty water controls (Table S2).

The diversity represented within each type of sample also varied (Table S4). Real bath toys showed on average a higher richness and more variation than control bath toys, ranging from 192–6196 OTUs per bath toy (1506 ± 1776, *n* = 17) compared to 188–268 (242 ± 33.9, *n* = 6) in the controls. This is also reflected with higher Shannon–Wiener indices for real bath toys (0.42 ± 0.13, *n* = 17), compared to both clean (0.22 ± 0.04, *n* = 3) and dirty water controls (0.28 ± 0.04, *n* = 3).

#### Most abundant bacterial OTUs in real and control bath toy biofilms

**Fig. 4:**
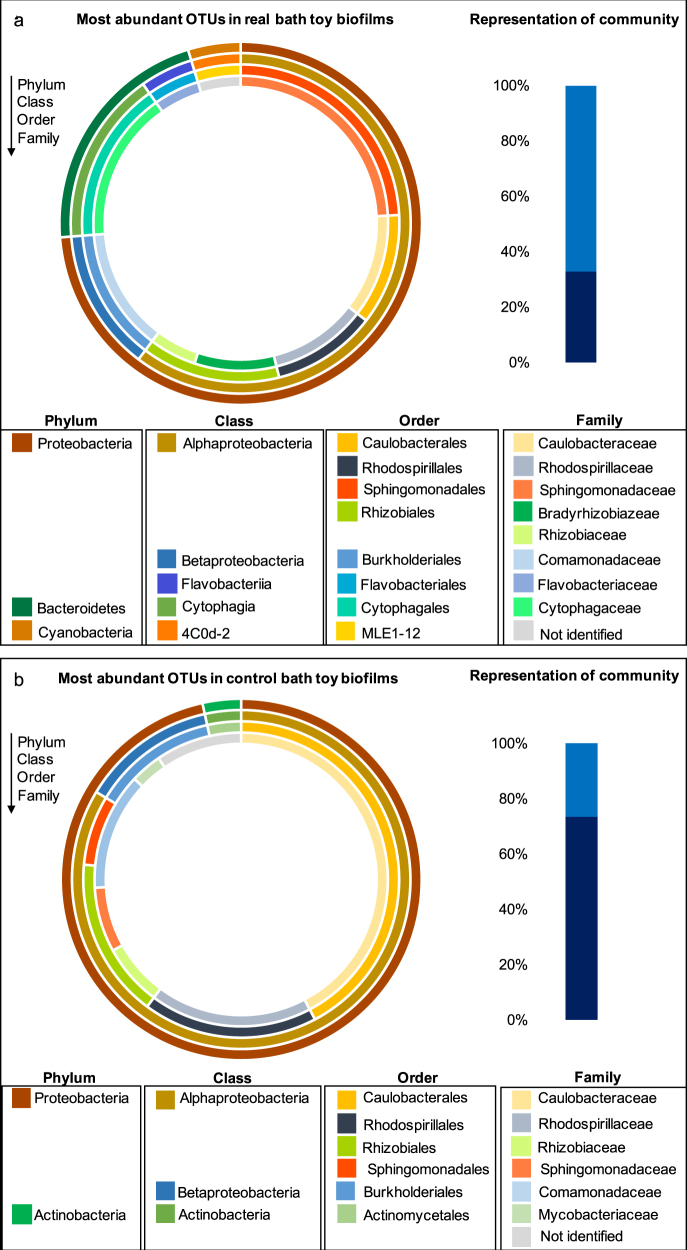
Classification of the ten most abundant operational taxonomic units (OTUs) for biofilm communities grown in real bath toys (**a**) or control bath toys (**b**). Outer to inner circles represent classifications from phylum to family level (the lowest common classification level). Bar plots represent the fraction of most abundant OTUs (dark blue bars) in comparison to the rest of the community (light blue bars)

Due to the variable community compositions throughout real bath toy biofilms and among the controls, communities were characterized in more detail by focusing on the most abundant OTUs in each community. For this, OTUs with the highest number of reads within all bath toys of one category, namely real bath toys, clean water controls, and dirty water controls, were chosen (for a detailed identification of the most abundant OTUs in each individual real bath toy see Figure S5). The phylum Proteobacteria was the most abundant in real bath toy biofilms, followed by Bacteroidetes and Cyanobacteria (Fig. 4a). On family level (which was the lowest common level of classification), these most abundant OTUs were representatives of Comamonadaceae, Bradyrhizobiaceae, Caulobacteraceae, Sphingomonadaceae, Cytophagaceae, Rhizobiaceae, and Flavobacteriaceae; which, amongst others, have previously been identified in drinking water systems and corresponding biofilms^35–41^ or in fresh water systems (Rhodospirillaceae^42^). Some representatives of the families Comamonadaceae, Bradyrhizobiaceae, Sphingomonadaceae have previously been detected in human microbiota (e.g., gastrointestinal, oral, skin, airways).^43–45^ It should be noted that the ten most abundant OTUs compiled from the data of all real bath toys were not necessarily representative of individual biofilms (Fig. 4a). In fact, these OTUs represented on average 33% of the total number of reads, ranging 6–52% (33 ± 16.5%, *n* = 17) in individual bath toys, thus highlighting the diversity amongst all real bath toy biofilm communities. Interestingly, most of the identified abundant families in the Proteobacteria clade were identical between real and control bath toys, whereas the abundance of Actinobacteria, in particular the family Mycobacteriaceae, was only identified for control bath toys. In contrast to the real bath toys, the most abundant OTUs in control toys were considerably more representative of their community compositions, representing 73% (±7.8, *n* = 6) of individual communities (Fig. 4b), hence revealing less diversity compared to real bath toy biofilms.

#### Bathing events affect bacterial community composition in bath toy biofilms

**Fig. 5:**
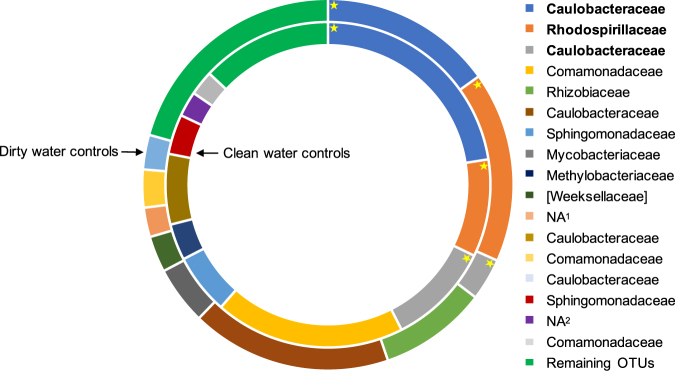
Comparison of the ten most abundant OTUs in control bath toy biofilms classified on family level. The inner circle represents bath toys used with clean water prior to bathing (clean water controls). The outer circle shows their composition in bath toys used with water after bathing (dirty water controls). Asterisks highlight the OTUs (in bold) that were abundant in both clean and dirty water controls. Each section of the plot represents the average relative abundance with the highest number of reads from triplicate bath toys. The portion of remaining OTUs is shown to emphasize the dominance of the ten most abundant OTUs for both clean and dirty water controls. OTUs are listed on family level as lowest common classification level, whereas “NA” represents OTUs that could only be classified on higher levels: NA^1^—Class of TM7-3, NA^2^—Order of Phycisphaerales. For further descriptions on the OTUs’ origins see Table S5

In control bath toy biofilms, the ten most abundant OTUs were representative for the total community composition (see above). For clean water controls, the ten most abundant OTUs covered 87 ± 2.6% (*n* = 3) of the total community composition, while those for dirty water controls accounted for 79 ± 9.9% (*n* = 3) (Fig. 5). Hence, we compared the most abundant OTUs of clean and dirty water control toys in more detail. Here, comparisons focus on families as this was the lowest common level for most of the OTUs (for deeper classification levels see Table S5). In clean water controls, nine out of the ten most abundant OTUs were members of Proteobacteria and only one belonged to the phylum Planctomycetes. For dirty water controls, the ten most abundant OTUs were more diverse, with seven belonging to Proteobacteria and one each in Bacteroidetes, Actinobacteria, and TM7 respectively (Table S5). Comparing these dominant OTUs of both control groups, only three out of ten were abundant in both clean and dirty water controls; namely members of the families Caulobacteraceae and Rhosospirillaceae (Fig. 5). The majority of identified families correlated with ones identified for real bath toy biofilms (Fig. 4). Other OTUs belonged to the families Methylobacteriaceae, Mycobacteriaceae, and, on a higher classification level, to the order Phycisphaerales, all of which have been detected in drinking or fresh water systems as well.^28,46–48^ Even amongst these samples with controlled conditions, results varied. To measure reproducibility, standard deviations of the percent community represented by the ten most abundant OTUs for the triplicate control bath toys were calculated. The deviations ranged immense from 0.9–157% in clean water controls and 6.3–164% in dirty water controls (data not shown). These variations between triplicates emphasize differences in the natural assembly of those biofilm communities under even identical conditions.

#### Fungi identified in studied bath toy biofilms

Fungal species could be identified in 11 out of 19 real bath toy biofilms, in all dirty water controls, and none of the clean water controls (Figure S6). Overall, the fungal communities were dominated by the phylum Ascomycota, the largest phylum of fungi. For real bath toy biofilms, the most abundant OTUs were representatives of the genera *Exophiala* spp., *Phialophora* spp., and *Fusarium* spp., all of which have previously been detected in drinking water systems.^49–51^ *Verticillum* spp. on the other hand is most commonly found in soil.^52^ For dirty water controls, not only Ascomycota but also the phylum Basidiomycota was represented (Figure S6). For the first, the genus *Scolecobasidium* spp. was abundant in two out of the three dirty water controls. Members of this genus have been identified in soil samples but also in environments like bath rooms.^53^ *Cryptococcus* spp., a representative of Basidiomycota, has been detected in spring, surface, and ground waters.^54^ Finally, *Polyporales* spp. is known to be an important fungal genus in forest ecosystems.^55^

### Presence of potentially harmful microorganisms in bath toy biofilms

#### Presence of culturable indicator bacteria and opportunistic pathogens in bath toy biofilms

In total, 80% of all studied bath toy biofilms showed positive cultivation results for at least one indicator organism or potential opportunistic pathogen [see Table S6 for numbers of colony-forming units (CFU)/bath toy]. The majority of real bath toy biofilms (61%) tested positive for *P. aeruginosa*. *Listeria* spp. and *L. pneumophila* were identified in 33%, while *Enterococci* spp. was present in 22% of real bath toy biofilms. As for control bath toys, all of the sampled biofilms tested positive for both *P. aeruginosa* and *Listeria* spp. Additionally, 66.6% of the clean and 100% of the dirty water controls showed positive results for *L. pneumophila*. Interestingly, coliforms (excluding *E. coli*) were only detected in clean water controls, while *E. coli* specifically was only found in dirty water controls. It should be noted that positive colonies were not subjected to additional confirmation tests.

#### Presence of genera of concern based on sequencing data

Sequencing data were further analyzed for genera of concern, including the bacteria analyzed with cultivation (above). *Pseudomonas* spp. could be identified on genus-level for all examined bath toys, ranging between 0.007 and 4.8% of total reads. Enterobacteriaceae was detected in 47% of the real bath toy samples, 67% of the clean, and 33% of the dirty water controls (ranging between 0.003–2.6% of total reads). In one real bath toy (Toy04), reads for *Klebsiella* spp., a genus of Enterobacteriaceae, made up 4.8% of the total number of reads of the biofilm community. *Klebsiella* spp. is not only relevant due to its presence in drinking water systems and biofilms, but also as a fecal indicator.^56–58^ Legionellaceae was identified in 94% of the real bath toy biofilms, in 66.7% of the clean, and 100% of the dirty water controls (0.01–0.53% of total reads). Listeriaceae could not be identified from the sequencing data. However, interestingly, the presence of *Staphylococcus* spp., which is a known pathogenic relative of *Listeria* spp., could be detected in 47% of the real bath toy biofilms and in one of each control bath toys (0.003–0.1% of total reads). Also, *Streptococcus* spp., an indicator for fecal contamination,^59,60^ was identified in 50% of all real bath toys, with 0.01–0.64% of the total number of reads. In addition, *Mycobacterium* spp., known for its presence in potable water and building plumbing installations, as well as for severe diseases,^28,46^ was present in all but one real bath toy biofilms and all dirty control toys, with a wide range of 1.19 ± 2.11% (*n* = 18) in real bath toys and 5.26 ± 2.19% (*n* = 3) in dirty water controls. Finally, *Chlamydia* spp. as well as *Clostridia* spp. could be identified in five real bath toys with 0.06 or 0.02%, respectively, with *Clostridia* spp. being an indicator for drinking water contamination,^61^ and *Chlamydia* spp. being part of the human, e.g., oral microbiome but also for representatives causing diseases.^62,63^

#### Identification of potentially harmful fungal groups based on sequencing data

Regarding the presence of potentially harmful fungal groups, the majority of the real bath toy biofilms showed positive results for *Exophiala*, members of which are potential agents of human and animal mycoses.^64^ The genus *Phialophora* spp. was also identified for some real bath toys, being a member of the “black yeast and relatives”, among which *P. verrucosa* has been reported to cause human infections.^65^ Finally, infections by *Fusarium* spp. have been reported,^66^ with *F. solani* being one of the main pathogenic relatives.^67^ Samples of the dirty water controls showed representatives of *Cryptococcus* spp., which include several important human pathogens, such as *Cryptococcus neoforman*.^68^

## Discussion

Almost one decade ago, the potential chemical risks of bath toys were documented in the colorfully titled book “Slow death by rubber duck”.^69^ In contrast, little scientific information is available on microbial colonization and risks in these bath toys, even though related esthetic and potential hygienic problems have been recognized in social media (Table S1). Therefore, the goals of this study were firstly to characterize biofilms grown on the inner surfaces of real bath toys and secondly to understand factors influencing biofilm growth and community composition using control bath toys. Based on these data we argue that the combination of four main factors impacted the magnitude and composition of bath toy biofilms, namely (1) the flexible plastic material and (2) the bath water quality that is further influenced by (3) chemical additives from washing products and the user, plus (4) biological contamination by the user’s microbiome and the environment.

### Flexible plastic material supports microbial growth

Bath toys are made from flexible synthetic polymeric materials, mostly polyvinyl chloride (PVC) or silicone rubber^69^ (https://www.badeenten.de/badeenten-quietscheenten/; http://www.toyhalloffame.org/toys/rubber-duck). Research by Zobell showed that plastic materials adsorb some organic matter, which in turn enables biofilm formation, and which is highly dependent on the type of plastic material.^9^ Moreover, flexible polymeric materials are generally known to release a considerable amount of organic carbon compounds, which favor microbial growth and biofilm formation.^16,18,20^ These migrating compounds are typically not the primary polymers, but rather additives, such as plasticizers and stabilizers.^10–13^ In this study, the material composition of the real bath toys was not determined, nor were they tested for the amount of leaching AOC. The reason was that the real bath toys were all used for extended time periods and most migration evidently occurred prior to our sampling. Therefore, interpreting the impact of specific materials on biofilm formation and community compositions was not possible for real bath toy biofilms. In contrast, the control bath toys were all identical and their material was tested for both carbon migration potential (MP) and biomass formation potential (BFP) using the BioMig assay proposed by Wen and colleagues.^16^ This assay revealed a MP of 3.92 ± 0.27   µg total organic carbon (TOC)/cm^2^/day (*n* = 3) (Table S7), and a BFP of 6.6 ± 1.1 × 10^8 ^  cells/cm^2^ (*n* = 3) (Table S7). These values are high and comparable to BFP values measured for materials such as PVC-P (2.7 × 10^8^ cells/cm^2^) and 2% EPDM (8.4 × 10^8^ cells/cm^2^),^16^ as well as for some flexible shower hoses (2.9–8.3 × 10^8^ cells/cm^2^).^5^ In comparison, other studies showed lower BFP for PE-X_a_ and PE-X_b_ materials with values ranging between 3.4–4.6 × 10^7^ cells/cm^2^.^16,70^ In general, migration is dominant in new materials and diminishes over time,^15,16^ and therefore, it is more relevant in new bath toys (i.e., control toys). However, it should be noted that BioMig assays are carried out under optimal conditions (e.g., with trace nutrient addition) for both carbon migration and BFP. Therefore, it is not surprising that values for biofilm coverage in the control bath toys (0.05–0.73 × 10^8^ cells/cm^2^) were on average lower than the predicted numbers with the BioMig assays (above). Importantly, the clean water controls showed significantly lower numbers than the dirty water controls (Fig. 2). Since the carbon migration by the material was identical in all control toys, we argue that differences in water quality caused the differences in cell numbers and community composition of clean and dirty water controls (discussed further below).

### Water quality influences microbial growth

#### Tap water quality

One seeding source for the microbial community of bath toy biofilms is the microbiome of the tap water. In this study, the real bath toys originated from five different households where water quality was not measured. Nevertheless, differences in the tap water microbiota can be expected,^22^ potentially causing variations in biofilm community compositions (e.g., Douterelo and colleagues^23^), and comprising household specific core communities. These differences occur due to several facts: (1) differences in source waters and treatment procedures dictate potable water communities,^71,72^ (2) spatial and temporal changes result in localized microbial biogeography,^73^ (3) differences in water heater temperatures and water usage frequencies influence the potable water microbiome,^74,75^ and finally, (4) the usage of different materials selects for individual microbial communities in the building plumbing system.^76,77^ Besides, several studies showed that microbial communities of potable water systems comprise opportunistic pathogens (e.g.,^78^), such as *L. pneumophila*, *P. aeruginosa*, *Mycobacterium* spp.^26,79^, or non-tuberculous mycobacteria.^24,28^ Thus, it’s not surprising that we recorded positive results for some of these organisms and/or genera to which they belong in several bath toy biofilms (Table S6; See above). As for nutrients, tap water poses an oligotrophic environment,^29,30,35,80,81^ with nutrient concentrations insufficient to support the degree of microbial growth observed in the bath toys (Table S8). Similar to the material, the tap water was identical for all control toys, suggesting that observed differences between clean water and dirty water controls (Fig. 2 and Fig. 3) are attributed to compounds and organisms associated with the dirty bath water.

#### Additional nutrients support bacterial growth

Under normal use conditions, bath toys are exposed to dirty bath water. This comprises additional organic and inorganic nutrients,^32^ which were shown to be biodegradable^82^ and thus beneficially impacting microbial growth.^83^ For example, Blackstock and colleagues showed that not only personal care products, but also body fluids like urine and sweat contribute to the amount of dissolved organic carbon (DOC) in the water.^33^ Urine in particular is a source for additional nitrogenic compounds in the form of urea, ammonia, or amino acids.^33,84^ In the control experiment of this study, chemical analysis of the water before and after bathing showed that concentrations in DOC and TOC increased ten-fold, while total nitrogen and phosphorous concentrations were doubled after a bathing event (Table S8). These results explain the higher biofilm coverage in dirty water controls compared to clean water controls (Fig. 2), despite the identical growth potential of the plastic material (Table S7) and identical tap water (above). However, it is not possible to conclude from the data whether the growth in the dirty water controls were driven predominantly by the additional organic nutrients or by the inorganic nutrients that enabled optimal use of carbon migrating from the plastic material.

#### Additional microbial contamination by the human end-user

In addition to the nutrient supply, dirty bath water also serves as a further source of microbial seeding for the bath toys, including both human microbiota and environmental bacteria that are released during bathing.^3,4,22,31,85^ For bacterial numbers, an increase of total cell concentration (TCC) from 2.1 ± 1.1    × 10^5^ to 4.1 ± 2.5    × 10^5^ cells/mL (*n* = 2) could be shown in the tap water after bathing (Table S8). In this study, the microbiome of users and/or their bath tubs were not sampled due to (1) privacy concerns for the children involved and (2) because a single grab sample would only have been representative for one particular time frame and not the period (often multiple years and multiple users) of use/exposure. The human microbiome was previously shown to differ between people. For example, a previous study noted that armpits of different individuals show clear differences in bacterial community compositions, which amongst others could be explained by the use of different care products, e.g., deodorants.^86^ The same applies for the microbiome colonizing other parts of the human skin, e.g., forearms,^87,88^ as well as the gut microbiome which depends on peoples age, health, and diet.^89–91^ With this multiplicity, potentially harmful bacteria can get released into the bath water as well. It was previously shown that dirty bath water contains significant amounts of, e.g., *E. coli* or other fecal coliforms,^31,32,82,92,93^ which supports our cultivation and sequencing data showing organisms/genera of concern in many bath toy biofilms. As for the input of environmental bacteria into the bath water, their origin strongly depends on the activities taken by the person bathing, e.g., soil bacteria after playing in the garden or limnic bacteria after swimming in a lake. It is evident that the pioneer organisms for the biofilm communities in clean water controls were predominantly the microbiome of the tap water and microorganisms potentially present in the clean bath tub, while dirty water controls were additionally influenced by (1) the human microbiome and (2) bacteria from the environment. However, our data did not allow differentiation between the contribution of these two. The points above in turn also explain the low abundance in real bath toy “household-specific core communities”, with different/multiple users, variations in environmental contamination and also variations in patterns (e.g., frequency of use) most likely contributing to the selection in the biofilm communities.

### Implications for the end-user

Environmental exposure to bacteria and fungi is not necessarily bad for human health and may indeed even strengthen the immune defense. Nevertheless, two studies have already shown the clinical relevance of bath toy biofilms.^2,34^ While we identified several indicator organisms and genera of concern, the data from both cultivation and sequencing have to be interpreted carefully. Cultivation data can be biased, e.g., due to potential non-selective growth of non-targeted organisms. Similarly, OTUs associated with genera of concern are not necessarily representatives of (opportunistic) pathogens, as strain-level classification was not possible with this approach. Nevertheless, bath toys are typically used by children, who are potentially sensitive and vulnerable users. Squeezing water with chunks of biofilm into their faces (which is not unexpected behavior for these users) may result in eye, ear, wound or even gastro-intestinal tract infections. To assess the real extent of this risk, more experimental work with specific focus on hygienic aspects is needed. Meanwhile, there are plenty of recommendations for cleaning and storing bath toys (e.g., boiling, removing water after usage) to minimize the risk of infection (Table S1). In addition, one could argue for increased regulations on polymeric materials used for bath toy production. This has already been done with respect to toxic chemical substances,^16,69^ while comprehensive material tests with respect to migration and microbial growth potential are available and increasingly used for building plumbing materials control.^94^ In fact, the easiest way to prevent children from being exposed to bath toy biofilms is to simply close the hole—but where is the fun in that?

## Conclusions

Bath toys from real households are colonized by dense biofilms with complex bacterial and fungal communities. Following the comparison of biofilms grown in clean and dirty water controls, we concluded that the coverage as well as the composition of these biofilm communities depended on the combination of four main factors namely: (1) the flexible plastic material that is releasing AOC and therefore favoring microbial growth; (2) the tap water microbiome that introduces specific microorganisms, potentially including opportunistic pathogens, to the bath toys; (3) additional nutrients in the dirty bath water due to personal care products and human body fluids; and (4) additional bacteria originating from both the user microbiome and environmental contamination. As this was a fundamental characterization study of such bath toy biofilms, further investigations for detailed risk assessment are needed.

## Materials and methods

### Bath toy samples

We collected 19 real bath toys (e.g., rubber ducks) from five different Swiss households, whereat the number of samples was determined by availablity. Due to privacy concerns of underage users, no specific information about the age of these bath toys or habits of use was collected. Moreover, no details about the bath toy producers, origin of materials, or water composition were compiled as all bath toys have been used for long time periods. For comparison, six bath toys were used under controlled conditions. These control bath toys were identical, purchased from a single batch, and used in an adult-only household over a period of 11 weeks with baths every second day. The control bath toys were divided into two categories—three bath toys (experimental replicates) were exposed to clean water before bathing, while three were exposed to the used bath water after bathing. The unused bath water was non-chlorinated groundwater and the used bath water was in all cases exposed to one adult using a commercially available soap product. Each control bath toy was filled three times with water, which was immediately squeezed out again, followed by a storage of the bath toys for two days on a shelf in the bathroom prior to reuse. After 11 weeks (equaling 39 exposures) the bath toys were transported to the laboratory, stored at 4 °C, and processed on the same day.

### Biofilm visualization

Each bath toy was cleaned on the outside with 70% ethanol and then dissected in half to access and characterize the biofilms on the inner surface. One half was used for biofilm analysis (below), while the other was photographed and used for further image analysis. The structure and thickness of selected bath toys’ biofilms were visualized with OCT, using a Spectral Domain OCT Imaging System (930 nm, OCT System Ganymede, Thorlabs GmbH, Dachau, Germany). Due to the heterogeneous biofilm distribution on the uneven toy surfaces, no data were collected for an overall quantification, but an approximate upper limit for biofilm thickness in analyzed bath toys could be set. For the visualization with SEM, 1 cm^2^ pieces of some real bath toys were chosen. Samples were fixed with 2.5% Glutaraldehyde in Cacodylate buffer (0.1 M, pH 7.2) for 1 h at room temperature, and thereafter stored in Cacodylate buffer at 4 °C. Final sample preparation and imaging was done by the Center for Microscopy and Image Analysis, University of Zurich.

### Biofilm removal

Biofilms were removed from the inner surface of bath toys using an electric toothbrush (Oral-B^®^, Advanced Power) as follows: one half of each bath toy was put into a sterile beaker and submerged in 100–150 mL ultrapure water. The biofilm was then removed by brushing the bath toys’ surface for approximately 2 min and this suspension was collected in 50 mL tubes (CellStar^®^ Tube, Greiner Bio-One). The whole procedure was repeated once with fresh ultrapure water to make sure all biofilm was removed. Biofilm clumps and clusters were subsequently dispersed with a sonication needle (Sonopuls HD 2200, Bandelin Sonorex, Rangendinen, Germany) for 30 s at 50% power and 40% intensity. Thereafter, the biofilm suspensions of one bath toy were combined in a sterile SCHOTT^®^ bottle and the volume was filled up with ultrapure water to a total volume of 500 mL. The toothbrush heads were replaced for each sample to avoid cross contamination.

### Flow cytometric cell counting

Flow cytometry (FCM) was used to determine the number of total and intact bacterial cells present in the biofilm suspensions. Measurements and analysis were performed as described elsewhere.^95^ In short, biofilm suspensions were diluted 1:100 with ultrapure water. Five hundred microliters of each sample were either stained with 5 µL SYBR^®^ Green I (SG, Invitrogen AG, Basel, Switzerland; 100× diluted in Tris buffer, pH 8) to detect the TCC or with 5 µL SG with additional propidium iodide (final concentration of 0.3 mM) to quantify the intact cell concentration. Prior to measurements, samples were incubated for 10 min at 37 °C. A BD Accuri C6^®^ flow cytometer (BD Accuri Cytometers, Belgium) was used, applying the same settings and gating strategy as described previously.^95^ All samples were measured in triplicate.

### Next generation sequencing for bacterial and fungal community compositions

MiSeq^®^ Sequencing (Illumina, Inc., San Diego, CA) was chosen to study the community compositions of the bath toy biofilms. For that, the biofilm suspensions were concentrated on 0.22 µm polycarbonate Nucleopore^®^ membrane filters (Ø 47 mm, Whatman, Kent, UK), using sterile filter units under vacuum pressure. The filtered volume was in all cases 490 mL (±5 mL). DNA was extracted according to the protocol of the PowerWater DNA Isolation Kit (MoBio Laboratories, Inc., Carlsbad, CA) and quantified with a Qubit^®^ 2.0 Fluorometer (Invitrogen, LT Holdings Pte Ltd, Singapore). For each sample, 1 ng of DNA was amplified by polymerase chain reaction (PCR) (for settings see Table S7), using Bakt_341F and Bakt_805R primers^96^ for the targeted V3-V5 region of the 16 S rDNA (final concentration 0.3 µM). Specific barcoded Nextera XT v2 Index Kit adapters (Illumina) were added to the amplicons via Index PCR (for settings see Table S7). The Agencort^®^ AMPure^®^ XP system (Beckman Coulter, Inc., Bera, CA) was performed after both amplification steps for purification. After successful amplification, PCR products were again quantified with a Qubit Fluorometer, followed by a normalization to concentrations of 4 nM (10 mM Tris, pH 8.0). Ten microliters of each normalized sample were pooled and thereafter quantified to ensure the final concentration. The sequencing was run at the MiSeq platform, adding 10% PhiX for quality control. Data for this community composition analysis was generated in collaboration with the Genetic Diversity Centre, ETH Zurich.

For DNA analysis, first, primer sites of all sequences were trimmed followed by merging overlapping reads. Second, sequences were filtered according to their quality, which was validated in a Quality report (FastQC v0.11.2). Finally, sorted reads were taxonomically assigned using QIIME with phylogenetic analysis for OTU sequences by PyNAST alignment. Here, a 97% identify cut-off as well as an abundance baseline of 2 was approached for clustering. Even though clustering based on a 97% similarity allows an identification as specific as genus level, this was not the case for most of the OTUs in this study. Hence, most of the community composition-based analyses focused on the lowest classification level that was common for most of them, which was family. Further data processing was conducted in RStudio (Version 0.99.902) using the packages “ggplot2” and “phyloseq”. All samples were scaled to an even minimum depth of 30,133 reads, which correlated to a total of 12,229 OTUs. Here, two real bath toy samples had to be excluded for further analysis due to low numbers of total reads. For DNA analysis of the fungal community composition, the ITS1 region of the extracted DNA was amplified using ITS1-F and ITS2 as described elsewhere.^97^ Two sets of amplicons from each sample were obtained with primers of different barcode sequences. Owing to the low concentration of genomic DNA, samples that failed to be amplified during the first two rounds were tested with increasing template concentrations in the PCR reaction for another three times. The resulted amplicons were pooled with equal amount (100 ng) after quantification with Qubit. The pool was purified using the Wizard SV Gel and PCR Clean-Up system (Promega, Madison, WI) and sequenced using MiSeq paired-end reads (2 × 250 bp) at the Roy J. Carver Biotechnology Center (University of Illinois at Urbana-Champaign). The paired-end reads were aligned with Mothur^98^ and were further analyzed using QIIME with the QIIME/UNITE reference OTUs (alpha version 12_11) and default parameters for demultiplexing, quality filtering, and clustering reads into OTUs.

### Conventional plating for specific bacterial groups

As children’s infections by opportunistic pathogens pose a big concern regarding bath toys, conventional plating was used as a proof of principle for their potential presence. Here, Compact Dry Plates (HyServe, Germany) were used to detect fecal indicator organisms and opportunistic pathogens; specifically, *Escherichia coli* and Coliforms (EC), *Pseudomonas aeruginosa* (PA), *Listeria* spp. (LS), and Enterococcus spp. (ETC). Special agar plates (Legionella BMP α Selective Medium, PO5035A; Oxoid, Thermo Fisher, Wesel, Germany) were used for the detection of *Legionella pneumophila* (LEG). In this case, the biofilm suspensions were heat shocked (55 °C, 30 min) to eliminate other bacteria prior to plating. One milliliter of the biofilm suspensions was added to each Compact Dry Plate or LEG plate in triplicate. The plates were incubated at 37 °C for either 24 h (EC, ETC), 48 h (PA, LS) or 14 d (LEG), before counting the colony-forming units (CFU). Here, colonies of distinct colors were counted following the manual. Only for EC plates, two different colors have been counted as assigned by the producer, distinguishing between coliforms and specifically *E. coli*.

### BioMig assay to determine the bioavailability of migrating carbon compounds

A standardized material test^16^ was used to assess the control bath toys for (1) the MP of organic carbon from the plastic material in contact with water and (2) the BFM, which relies on the migrated carbon. In short: for the MP, a surface area of 100 cm^2^ from the control bath toys’ material was incubated in filtered bottled mineral water (Evian, France), at 60 °C, over a period of 7 days. The water was exchanged every 24 h and the amount of TOC was measured after day 1, 3, and 7 (TOC-V_CPH_, SHIMADZU GmbH, Switzerland). The BFP was determined by incubating 1 cm^2^-pieces of the material in unfiltered bottled water, at 30 °C, for 14 days, continuously shaking at 90 rpm. The number of planktonic bacteria (pTCC) was measured with FCM as described above. The same was done for the number of bacteria in the biofilm (bTCC), with an anterior needle sonication to remove the biofilm from the material in 0.2-µm-filtered bottled water. Additionally, a growth-test was conducted to evaluate the general degradability of the released TOC. Therefore, water of the first migration period from the MP assay was used to determine the amount of AOC. The samples were inoculated with the natural microbial community of bottled water and incubated for 7 days at 30 °C, shaking. FCM was used to count the number of bacteria, followed by a re-calculation of the AOC-concentration needed for these cells to grow.^99,100^

### Data availability

DNA sequencing data is available at the European Nucleotide Archive (ENA) with accession number PRJEB24750.

## Electronic supplementary material

Supplementary Information(PDF 11004 kb)

## References

[1] Else TA, Pantle CR, Amy PS (2003). Boundaries for biofilm formation: humidity and temperature boundaries for biofilm formation: humidity and temperature. Appl. Environ. Microbiol..

[2] Ruschke R (1976). Kunststoff-Schwimmtiere als Biotop für Mikroorganismen und mögliche Infektionsquellen für Kleinkinder. Zbl. Bakt. Hyg. I. Abt. Orig. B.

[3] Finch JE, Prince J, Hawksworth M (1978). A bacteriological survey of the domestic environment. J. Appl. Bacteriol..

[4] Scott E, Bloomfieldt SF, Barlow CG (1982). An investigation of microbial contamination in the home. J. Hyg..

[5] Proctor CR (2016). Biofilms in shower hoses—choice of pipe material influences bacterial growth and communities. Environ. Sci. Water Res. Technol..

[6] Soto-Giron MJ (2016). Biofilms on hospital shower hoses: characterization and implications for nosocomial infections. Appl. Environ. Microbiol..

[7] Feazel LM (2009). Opportunistic pathogens enriched in showerhead biofilms. Proc. Natl. Acad. Sci..

[8] Kelley ST, Theisen U, Angenent LT, St Amand A, Pace NR (2004). Molecular analysis of shower curtain biofilm microbes. Appl. Environ. Microbiol..

[9] Zobell CE (1943). The effect of solid surfaces upon bacterial activity. J. Bacteriol..

[10] Zhang L, Liu S (2014). Investigation of organic compounds migration from polymeric pipes into drinking water under long retention times. Procedia Eng..

[11] Holsen TM, Park JK, Jenkins D, Selleck RE (1991). Contamination of potable water by permeation of plastic pipe. Am. Water Work Assoc..

[12] Skjevrak I, Due A, Gjerstad KO, Herikstad H (2003). Volatile organic components migrating from plastic pipes (HDPE, PEX and PVC) into drinking water. Water Res..

[13] Stern BR, Lagos G (2008). Are there health risks from the migration of chemical substances from plastic pipes into drinking water? A review. Hum. Ecol. Risk Assess. Int. J..

[14] Connell M (2016). PEX and PP water pipes: assimilable carbon, chemicals, and odors. J. Am. Water Works Assoc..

[15] Bucheli-Witschel M, Koetzsch S, Darr S, Widler R, Egli T (2012). A new method to assess the influence of migration from polymeric materials on the biostability of drinking water. Water Res..

[16] Wen G, Kötzsch S, Vital M, Egli T, Ma J (2015). BioMig—a method to evaluate the potential release of compounds from and the formation of biofilms on polymeric materials in contact with drinking water. Environ. Sci. Technol..

[17] Schoenen, D. & Schöler, H. Microbial alterations of drinking water by building materials—field observations and laboratory studies. *Water Quality and Technology Conference Proceedings, Houston, Texas,* 307–317 (1985).

[18] Lehtola MJ (2004). Microbiology, chemistry and biofilm development in a pilot drinking water distribution system with copper and plastic pipes. Water Res..

[19] Niquette P, Servais P, Savoir R (2000). Impacts of pipe materials on densities of fixed bacterial biomass in a drinking water distribution system. Water Res..

[20] Yu J, Kim D, Lee T (2010). Microbial diversity in biofilms on water distribution pipes of different materials. Water Sci. Technol..

[21] Kerr CJ, Osborn KS, Robson GD, Handley PS (1998). The relationship between pipe material and biofilm formation in a laboratory model system. J. Appl. Microbiol..

[22] Tamames J, Abellán JJ, Pignatelli M, Camacho A, Moya A (2010). Environmental distribution of prokaryotic taxa. BMC Microbiol..

[23] Douterelo I, Jackson M, Solomon C, Boxall J (2017). Science of the total environment spatial and temporal analogies in microbial communities in natural drinking water bio films. Sci. Total Environ..

[24] Parsek MR, Singh PK (2003). Bacterial biofilms: an emerging link to disease pathogenesis. Annu. Rev. Microbiol..

[25] Bodey GP, Bolivar R, Fainstein V, Jadeja L (1983). Infections caused by *Pseudomonas aeruginosa*. Rev. Infect. Dis..

[26] Falkinham J, Pruden A, Edwards M (2015). Opportunistic premise plumbing pathogens: increasingly important pathogens in drinking water. Pathogens.

[27] Fields BS, Benson RF, Besser RE (2002). Legionella and Legionnaire’s disease: 25 years of investigation. Clin. Microbiol. Rev..

[28] Falkinham JO (2011). Nontuberculous mycobacteria from household plumbing of patients with nontuberculous mycobacteria disease. Emerg. Infect. Dis..

[29] Boe-Hansen R, Martiny AC, Arvin E, Albrechtsen HJ (1989). Monitoring biofilm formation and activity in drinking water distribution networks under oligotrophic conditions. Water Sci. Technol..

[30] Boe-Hansen R, Albrechtsen HJ, Arvin E, Claus J (2002). Bulk water phase and biofilm growth in drinking water at low nutrient conditions. Water Res..

[31] Rose JB, Sun GS, Gerba CP, Sinclair NA (1991). Microbial quality and persistence of enteric pathogens in graywater from various household sources. Water Res..

[32] Eriksson E, Auffarth K, Henze M, Ledin A (2002). Characteristics of grey wastewater. Urban Water.

[33] Blackstock LK, Wang W, Vemula S, Jaeger BT, Li X (2017). Sweetened swimming pools and hot tubs. Env. Sci. Technol. Lett..

[34] Buttery JP (1998). Multiresistant *Pseudomonas aeruginosa* outbreak in a pediatric oncology ward related to bath toys. Pediatr. Infect. Dis. J..

[35] Martiny AC, Albrechtsen H, Arvin E, Molin S (2005). Identification of bacteria in biofilm and bulk water samples from a nonchlorinated model drinking water distribution system: detection of a large nitrite-oxidizing population associated with *Nitrospira spp*. Appl. Environ. Microbiol..

[36] Norton CD, LeChevallier MW (2000). A pilot study of bacteriological population changes through potable water treatment and distribution. Appl. Environ. Microbiol..

[37] Williams MM, Domingo JWS, Meckes MC, Kelty CA, Rochon HS (2004). Phylogenetic diversity of drinking water bacteria in a distribution system simulator. J. Appl. Microbiol..

[38] Zeng DN (2013). Analysis of the bacterial communities associated with different drinking water treatment processes. World J. Microbiol. Biotechnol..

[39] Kämpfer P, Lodders N, Busse HJ (2009). Arcicella rosea sp. nov., isolated from tap water. Int. J. Syst. Evol. Microbiol..

[40] Chao Y, Mao Y, Wang Z, Zhang T (2015). Diversity and functions of bacterial community in drinking water biofilms revealed by high-throughput sequencing. Sci. Rep..

[41] Koskinen R (2000). Characterization of Sphingomonas isolates from Finnish and Swedish drinking water distribution systems. J. Appl. Microbiol..

[42] Herbert Ra (1976). Isolation and identification of photosynthetic bacteria (Rhodospirillaceae) from Antarctic marine and freshwater sediments. J. Appl. Bacteriol..

[43] Ursell LK (2012). The interpersonal and intrapersonal diversity of human-associated microbiota in key body sites. J. Allergy Clin. Immunol..

[44] Mueller NT, Bakacs E, Combellick J, Grigoryan Z, Dominguez-Bello MG (2015). The infant microbiome development: mom matters. Trends Mol. Med..

[45] Rudney JD, Xie H, Rhodus NL, Ondrey FG, Griffin TJ (2010). A metaproteomic analysis of the human salivary microbiota by three-dimensional peptide fractionation and tandem mass spectrometry. Mol. Oral Microbiol.

[46] Gallego V, García MT, Ventosa A (2005). Methylobacterium hispanicum sp. nov. and Methylobacterium aquaticum sp. nov., isolated from drinking water. Int. J. Syst. Evol. Microbiol..

[47] Furuhata K (2006). Isolation and identification of methylobacterium species from the tap water in hospitals in Japan and their antibiotic susceptibility. Microbiol. Immunol..

[48] Vaerewijck MJM, Huys G, Palomino JC, Swings J, Portaels F (2005). Mycobacteria in drinking water distribution systems: ecology and significance for human health. FEMS Microbiol. Rev..

[49] Göttlich E (2002). Fungal flora in groundwater-derived public drinking water. Int. J. Hyg. Environ. Health.

[50] Nishimura K, Miyaji M, Taguchi H, Tanaka R (1987). Fungi in bathwater and sludge of bathroom drainpipes. Mycopathologia.

[51] Lian X, de Hoog GS (2010). Indoor wet cells harbour melanized agents of cutaneous infection. Med. Mycol..

[52] Hirsch PR, Atkins SD, Mauchline TH, Morton CO, Davies KG, Kerry BR (2001). Methods for studying the nematophagous fungus Verticillium chlamydosporium in the root environment. Plant Soil.

[53] Abe N, Hamada N (2011). Molecular characterization and surfactant utilization of Scolecobasidium isolates from detergent-rich indoor environments. Biocontrol. Sci..

[54] Pereira VJ (2009). Occurrence of filamentous fungi and yeasts in three different drinking water sources. Water Res..

[55] Zhou, J., Zhu, L., Chen, H. & Cui, B. Taxonomy and phylogeny of polyporus group melanopus (Polyporales, Basidiomycota) from China. *PLoS One* https://doi.org/10.1371/journal.pone.0159495 (2016). 10.1371/journal.pone.0159495 PMC4972403 27486931

[56] Edberg SC, Rice EW, Karlin RJ, Allen MJ (2000). Escherichia coli: the best biological drinking water indicator for public health protection. J. Appl. Microbiol..

[57] Jeffrey JB, Xu HS, Colwell RR (1991). Viable but nonculturable bacteria in drinking water. Appl. Environ. Microbiol..

[58] Jeffrey GS, Rice EW, Bishop PL (2006). Persistence of Klebsiella pneumoniae on simulated biofilm in a model drinking water system. Environ. Sci. Technol..

[59] Spaander P, Roest ACF (1958). The detection of faecal Streptococci in drinking-water. Antoine Van Leeuwenhoek.

[60] Pinto B, Pierotti R, Canale G, Reali D (1999). Characterization of ‘ faecal streptococci’ as indicators of faecal pollution and distribution in the environment. Lett. Appl. Microbiol..

[61] Payment P, Franco E (1993). Clostridium perfringens and somatic coliphages as indicators of the efficiency of drinking water treatment for viruses and protozoan cysts. Appl. Environ. Microbiol..

[62] Dewhirst FE (2010). The human oral microbiome. J. Bacteriol..

[63] Miyairi I (2012). Host genetics and chlamydia disease: prediction and validation of disease severity mechanisms. PLoS One.

[64] Zeng JS (2007). Spectrum of clinically relevant exophiala species in the United States. J. Clin. Microbiol..

[65] Li Y (2017). Biodiversity and human-pathogenicity of Phialophora verrucosa and relatives in Chaetothyriales. Persoonia.

[66] Hennequin C (1999). Identification of Fusarium species involved in human infections by 28S rRNA gene sequencing. J. Clin. Microbiol..

[67] Guarro J, Gené J (1992). Fusarium infections. Criteria for the identification of the responsible species. Mycoses.

[68] Vilgalys R, Hester M (1990). Rapid genetic identification and mapping of enzymatically amplified ribosomal DNA from several cryptococcus species. J. Bacteriol..

[69] Smith, R. & Lourie, B. *Slow Death by Rubber Duck—The Secret Danger of Everyday Things. *(Counterpoint Press, Berkeley, California, 2009).

[70] Koetzsch S, Egli T (2013). Kunststoffe in Kontakt mit Trinkwasser. Aqua Gas.

[71] Pinto AJ, Xi C, Raskin L (2012). Bacterial community structure in the drinking water microbiome is governed by filtration processes. Env. Sci. Technol..

[72] Gomez-Smith CK, Tan DT, Shuai D (2016). Environmental Science water microbiome—from treatment to tap. Environ. Sci. Water Res. Technol..

[73] Roeselers G (2015). Microbial biogeography of drinking water: patterns in phylogenetic diversity across space and time. Environ. Microbiol..

[74] Ji, P., Rhoads, W. J., Edwards, M. A. & Pruden, A. Impact of water heater temperature setting and water use frequency on the building plumbing microbiome. *ISME J*. https://doi.org/10.1038/ismej.2017.14 (2017). 10.1038/ismej.2017.14 PMC5437349 28282040

[75] Ji P, Parks J, Edwards MA, Pruden A (2015). Impact of water chemistry, pipe material and stagnation on the building plumbing microbiome. PLoS One.

[76] Rozej A, Cydzik-Kwiatkowska A, Kowalska B, Kowalski D (2015). Structure and microbial diversity of biofilms on different pipe materials of a model drinking water distribution systems. World J. Microbiol. Biotechnol..

[77] Lin W, Yu Z, Chen X, Liu R, Zhang H (2013). Molecular characterization of natural biofilms from household taps with different materials: PVC, stainless steel, and cast iron in drinking water distribution system. Appl. Microbiol. Biotechnol..

[78] Williams MM, Armbruster CR, Arduino MJ (2013). Plumbing of hospital premises is a reservoir for opportunistically pathogenic microorganisms: a review. Biofouling.

[79] Szewzyk U, Szewzyk R, Manz W, Schleifer K (2000). Microbiological safety of drinking water. Annu. Rev. Microbiol..

[80] Wingender J, Flemming HC (2011). Biofilms in drinking water and their role as reservoir for pathogens. Int. J. Hyg. Environ. Health.

[81] van der Wende E, Characklis WG, Smith DB (1989). Biofilms and bacterial drinking water quality. Water Res..

[82] Li F, Wichmann K, Otterpohl R (2009). Review of the technological approaches for grey water treatment and reuses. Sci. Total. Environ..

[83] Miettinen IT, Vartiainen T, Martikainen PJ (1997). Phosphorus and bacterial growth in drinking water. Appl. Environ. Microbiol..

[84] Erdinger L, Kirsch F, Sonntag H (1997). Potassium as an indicator of anthropogenic contamination of swimming pool water. Zent. fur Hyg. und Umweltmed..

[85] Robinson CJ, Bohannan BJM, Young VB (2010). From structure to function: the ecology of host-associated microbial communities. Microbiol. Mol. Biol. Rev..

[86] Urban, J. et al. The effect of habitual and experimental antiperspirant and deodorant product use on the armpit microbiome. *Peer J*. 1–20 (2016). 10.7717/peerj.1605. 10.7717/peerj.1605 PMC4741080 26855863

[87] Kong HH (2011). Skin microbiome: genomics-based insights into the diversity and role of skin microbes. Trends Mol. Med..

[88] Grice EA (2008). A diversity profile of the human skin microbiota. Genome Res..

[89] Contreras M (2012). Human gut microbiome viewed across age and geography. Heal. Hum. Serv. USA.

[90] Lozupone CA, Stombaugh JI, Gordon JI, Jansson JK, Knight R (2012). Diversity, stability and resilience of the human gut microbiota. Nature.

[91] Shreiner AB, Kao JK, Young VB (2015). The gut microbiome in health and in disease. Curr. Opin. Gastroenterol..

[92] Chaillou K, Gérente C, Andrès Y, Wolbert D (2011). Bathroom greywater characterization and potential treatments for reuse. Water Air. Soil Pollut..

[93] Hourlier F (2010). Formulation of synthetic greywater as an evaluation tool for wastewater recycling technologies. Environ. Technol..

[94] Koetzsch S, Egli T (2013). Kunststoffe in Kontakt mit Trinkwasser. Aqua Gas.

[95] Prest EI, Hammes F, Kötzsch S, van Loosdrecht MCM, Vrouwenvelder JS (2013). Monitoring microbiological changes in drinking water systems using a fast and reproducible flow cytometric method. Water Res..

[96] Klindworth A (2013). Evaluation of general 16S ribosomal RNA gene PCR primers for classical and next-generation sequencing-based diversity studies. Nucleic Acids Res..

[97] Walters W (2015). Improved bacterial 16S rRNA Gene (V4 and V4-5) and fungal internal transcribed spacer marker gene primers. Methods Protoc. Nov. Syst. Biol. Tech..

[98] Kozich JJ, Westcott SL, Baxter NT, Highlander SK, Schloss PD (2013). Development of a dual-index sequencing strategy and curation pipeline for analyzing amplicon sequence data on the MiSeq. Appl. Environ. Microbiol..

[99] Liu W (2002). Investigation of assimilable organic carbon (AOC) and bacterial regrowth in drinking water distribution system. Water Res..

[100] Hammes FA, Egli T (2005). New method for assimilable organic carbon determination using flow-cytometric enumeration and a natural microbial consortium as inoculum. Env. Sci. Technol..

