# Supplementary material for: Ugly ducklings—the dark side of plastic materials in contact with potable water
Source: NPJ Biofilms Microbiomes. 2018 Mar 27;4:7. doi: 10.1038/s41522-018-0050-9 (PMC5869678; doi:10.1038/s41522-018-0050-9)
Supplement: Supplementary file 1 — Supplementary Information(PDF 11004 kb) [file 41522_2018_50_MOESM1_ESM.pdf]

**Supplementary Information**

**Ugly ducklings – The dark side of plastic materials in contact with potable water**

Lisa Neu[1,2], Carola Bänziger[1], Caitlin R. Proctor[1,2], Ya Zhang[3], Wen-Tso Liu[3], Frederik Hammes[1,*]

[1] Eawag, Swiss Federal Institute of Aquatic Science and Technology, Dübendorf, Switzerland

[2] Department of Environmental Systems Science, Institute of Biogeochemistry and Pollutant Dynamics, ETH Zürich, Zürich, Switzerland

[3] Department of Civil and Environmental Engineering, University of Illinois at Urbana-Champaign, USA

**Table of contents**

| | |
|---|---|
| **Table S1** | Exemplary online blog entries on biofouling in bath toys |
| **Figure S1** | Images of all examined bath toys |
| **Figure S2** | Additional images of bath toy biofilms by OCT |
| **Figure S3** | Additional images on biofilm composition by SEM |
| **Figure S4** | Number of bacteria and proportion of intact cells in bath toy biofilms |
| **Table S2** | Classification of shared OTUs between bath toys |
| **Table S3** | Shared and 'core' communities in bath toys from single households |
| **Table S4** | Richness and diversity |
| **Figure S5** | Classification of abundant OTUs in real bath toy biofilms |
| **Table S5** | Comparison of most abundant OTUs in control bath toy biofilms |
| **Figure S6** | Fungal community composition in bath toy biofilms |
| **Table S6** | Conventional plating results for indicator bacteria and groups |
| **Table S7** | Bioavailability of migrating carbon from control bath toys' material |
| **Water chemistry** | Method and results (**Table S8**) |
| **Table S9** | Settings for Amplification PCR and Index PCR reactions |

**Table S1:** Exemplary online blog entries on biofouling inside bath toys

| **Issue - What is the slime?** | **Link** |
|---|---|
| Rub-a-dub-dub, what's in the tub? | https://www.babble.com/baby/whats-in-the-tub/ |
| What's the black stuff in your squeeze toys? | http://blogs.babycenter.com/momstories/whats-the-black-stuff-in-your-squeeze-toys/ |
| Friday Find: NBC's Today Show segment: Do bath toys carry germs? | http://www.bebravekeepgoing.com/2010/03/friday-find-nbcs-today-show-segment-do.html |
| Yuck. Yuck. Yuck! (A.K.A. Why my kids no longer have rubber duckies!) | http://www.imperfecthomemaking.com/2012/11/yuck-yuck-yuck-aka-why-my-kids-no.html |
| | |
| **Issue – How to prevent or remove it?** | **Link** |
| How to clean bath toys & prevents mould | http://www.maids.com/blog/how-to-clean-and-prevent-mold-in-bath-toys/ |
| Glue gun the rubber ducky | http://lajollamom.com/glue-gun-the-rubber-ducky/ |
| How to remove mould from bath toys | http://www.howtocleanstuff.net/how-to-remove-mold-from-bath-toys/ |
| Ask Martha: How can I clean my children's bath toys? | http://www.marthastewart.com/1125723/cleaning-childrens-bath-toys |
| What's the best way to clean bath toys? | http://www.realsimple.com/magazine-more/inside-magazine/ask-real-simple/best-way-clean-bath-toys |

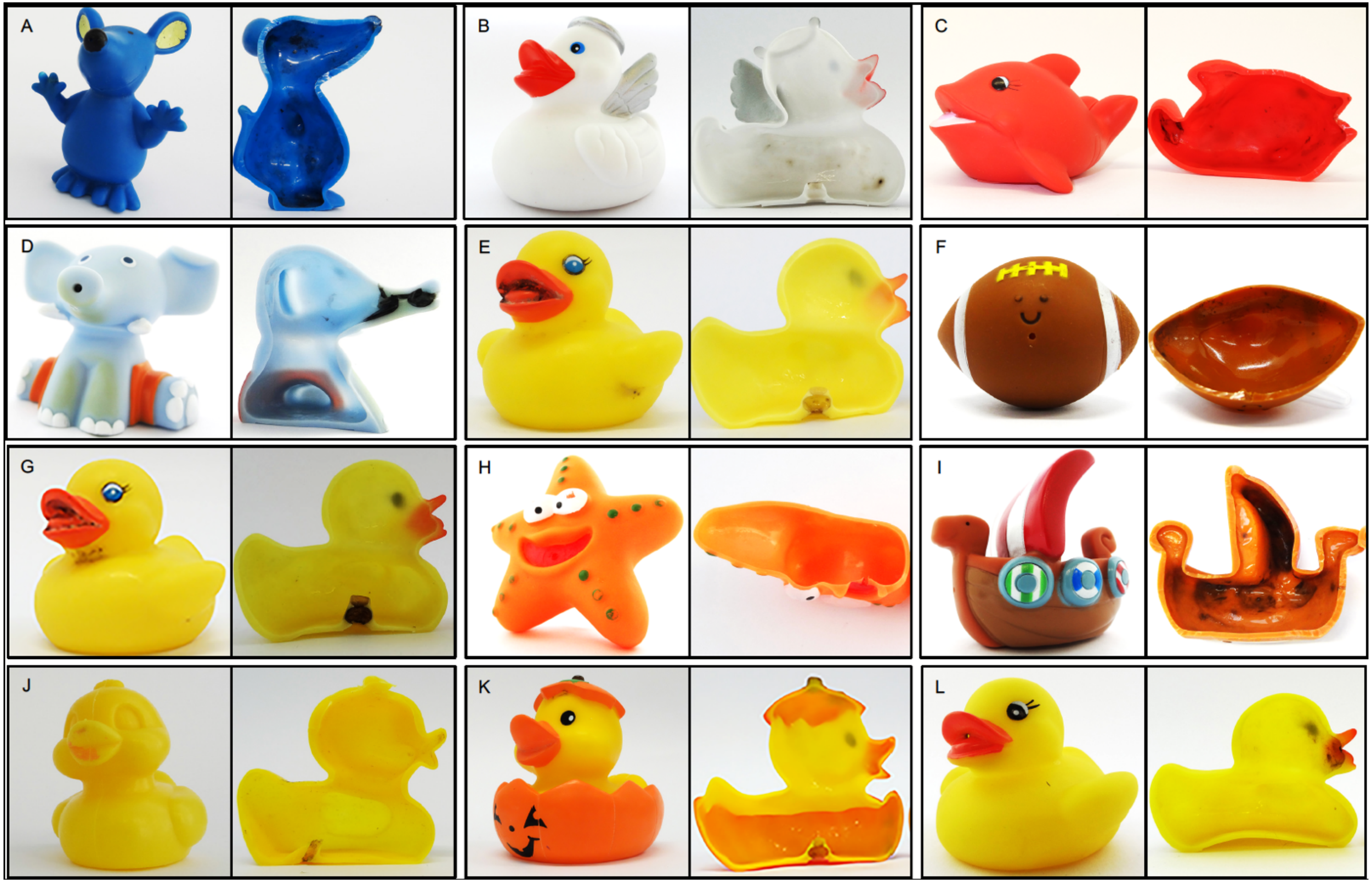


**Figure S1:** Images of all examined bath toys, both sound and cut open. A-L: bath toys from real households.

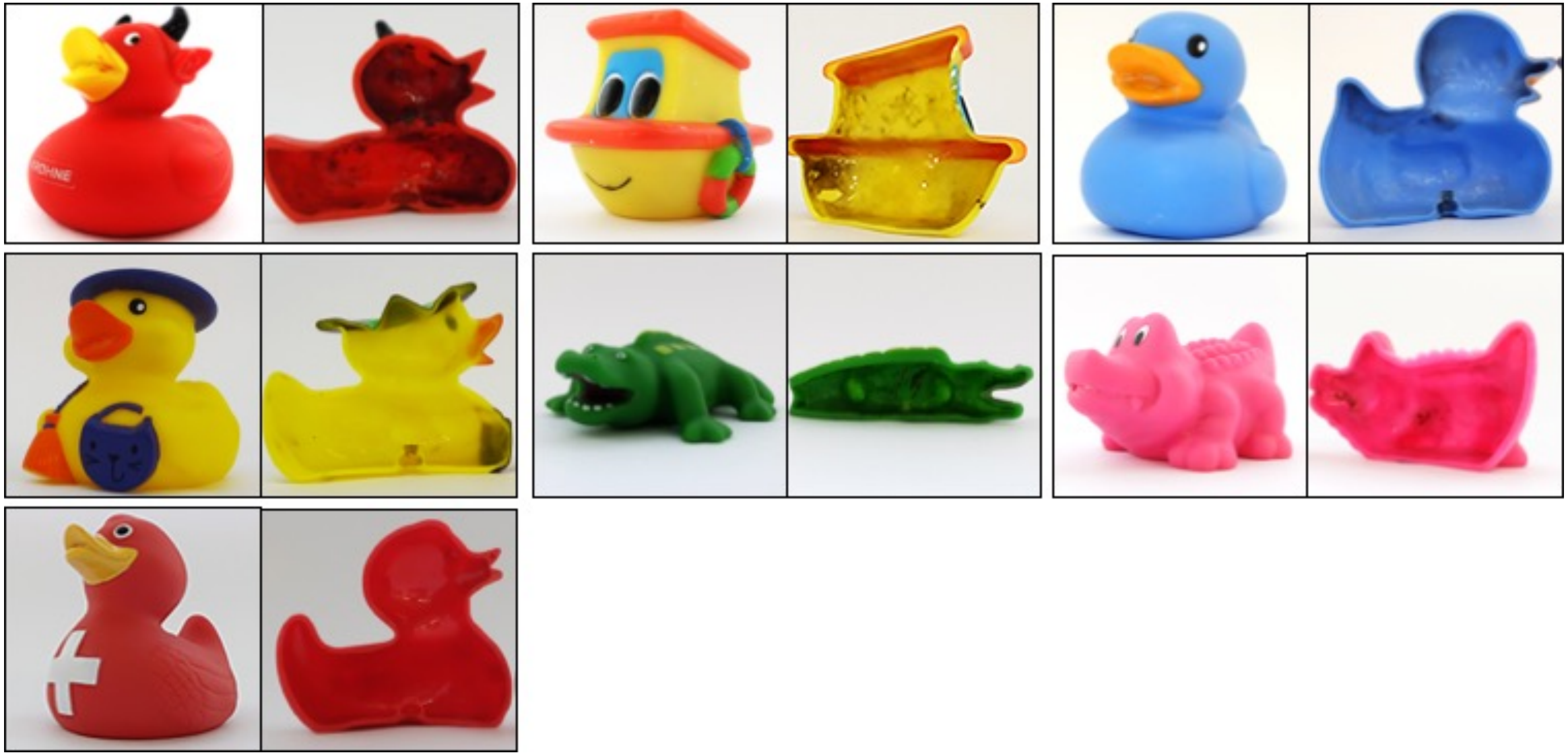

**Figure S1:** Images of all examined bath toys, both sound and cut open. M-S: bath toys from real households.

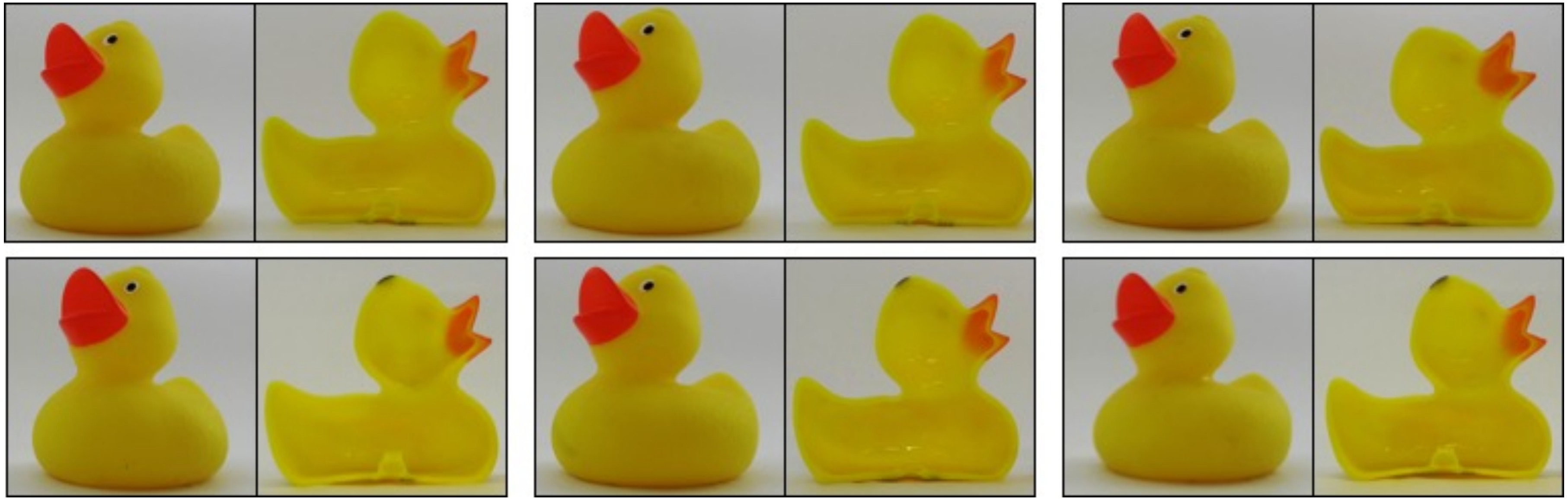

**Figure S1:** Images T-V: control bath toys processed with water prior to bathing (clean water controls). W-Y: control bath toys used with water after bathing (dirty water controls).

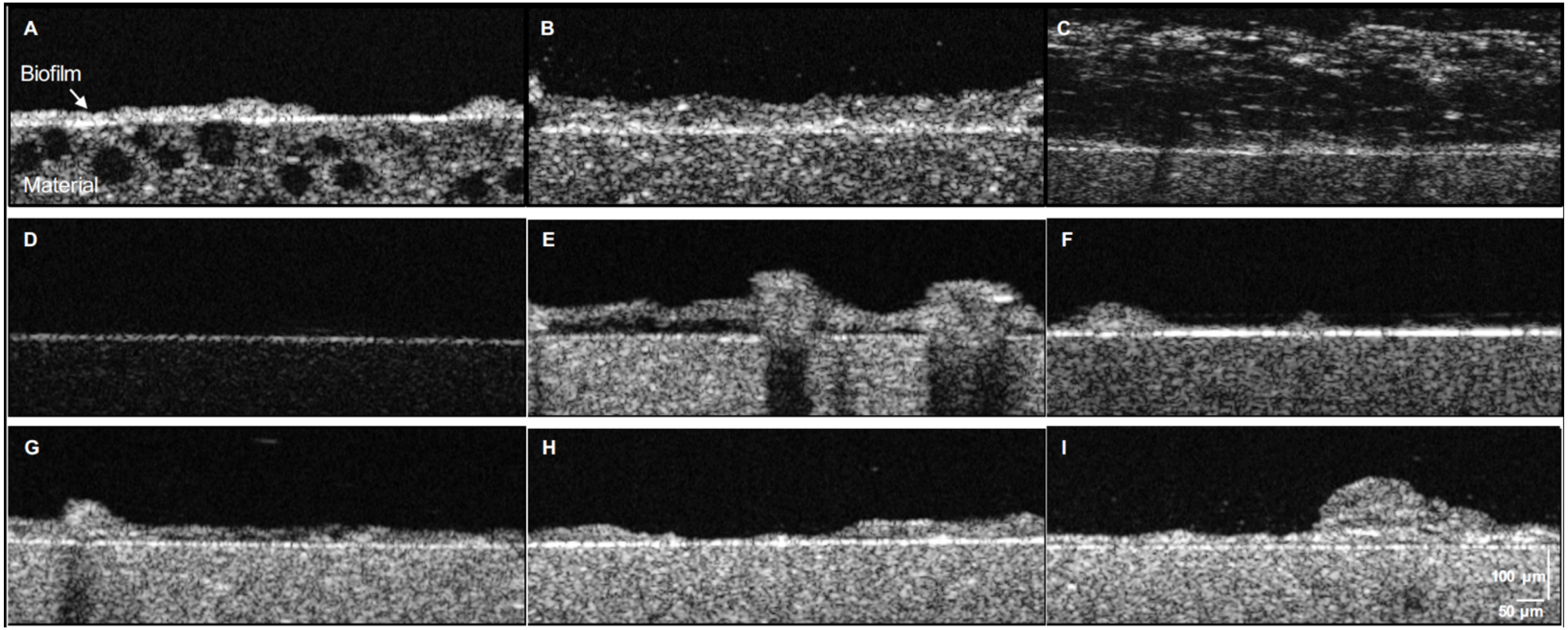


**Figure S2:** Optical coherence tomography (OCT) was used to image biofilm shape and thickness of selected bath toys. For real bath toys three exemplary biofilms are shown (A-C). Biofilms of clean water controls, which were used with water prior to bathing, are shown in D-F, while biofilms of dirty water controls, processed with water after bathing, are shown in images G-I.

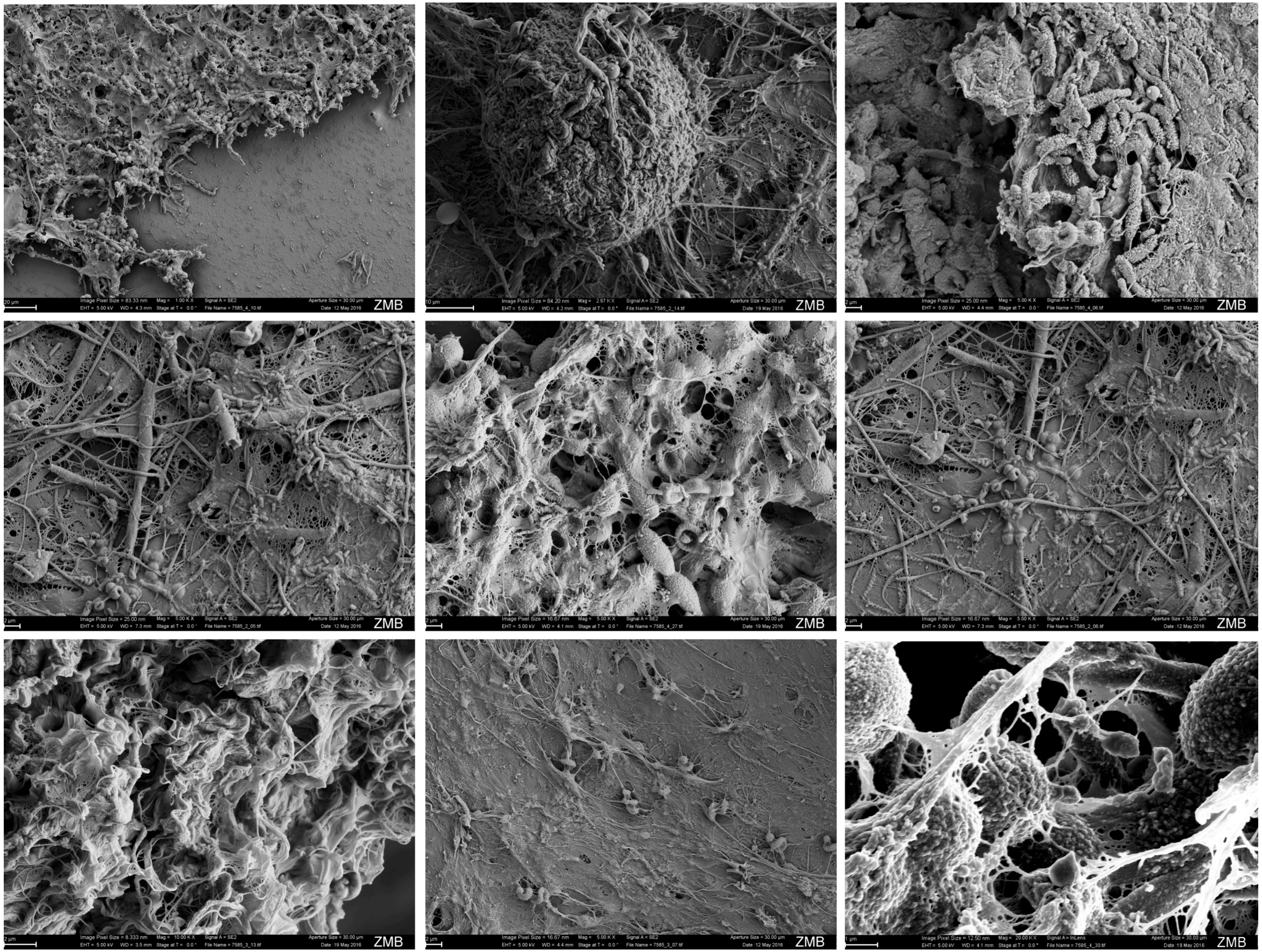


**Figure S3:** Additional images for biofilm composition by scanning electron microscopy. SEM was used to visualize the microbial community composition in some real bath toy biofilms. Images were taken by the Center for Microscopy and Image Analysis, University Zurich.

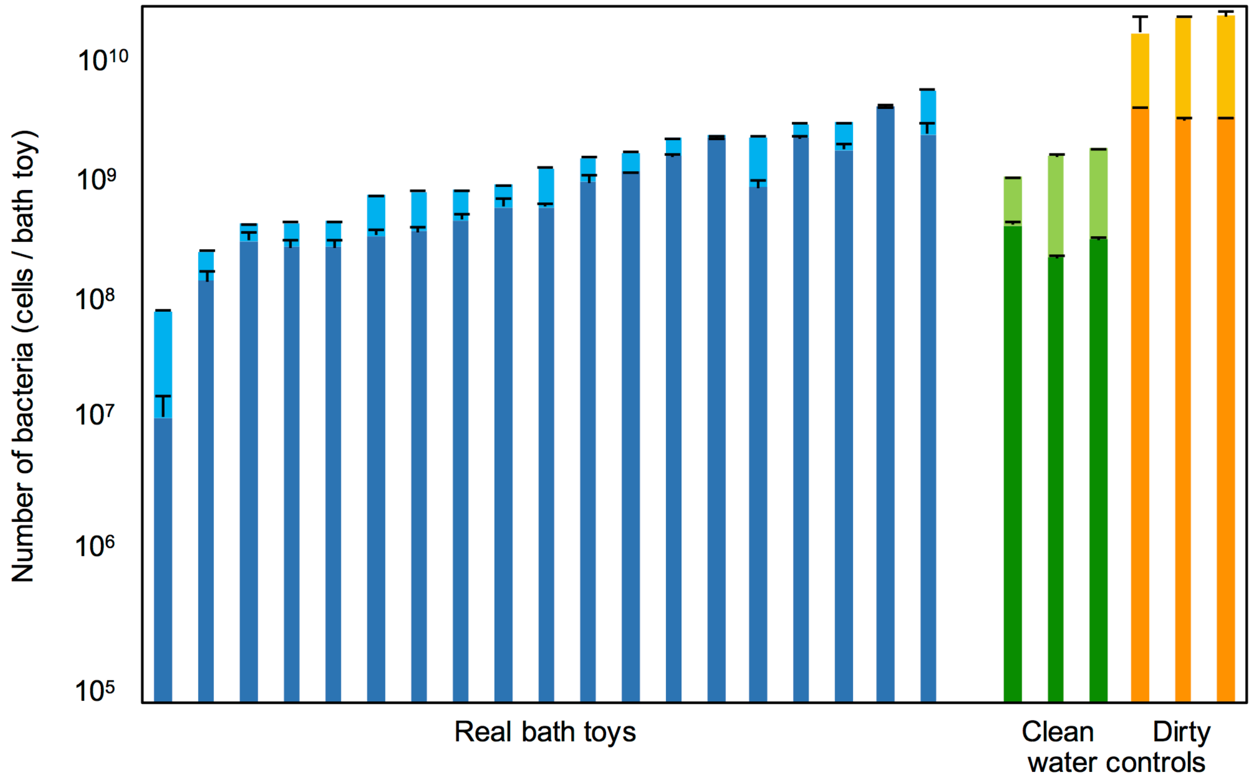


**Figure S4:** Number of bacteria in bath toy biofilms. Flow cytometry was used to differentiate between the total number of cells (SYBR® Green I staining) and the amount of intact cells (SYBR® Green I and Propidium Iodide staining) in biofilms grown in bath toys from real households or in control bath toys processed with either water prior (clean water controls) or after bathing (dirty water controls). The darker colours represent the number of intact bacteria in each bath toy, the lighter colours the damaged amount of the total number of cells. Error bars represent standard deviations of triplicate measurements.

Table S2: Classification of operational taxonomic units (OTUs) shared by biofilms originating from all bath toys ( ), all real bath toys ( ), all control bath toys ( ), all clean water controls ( ), or all dirty water controls ( ). NA indicates that no further classification could be made for that. “OTU-ID name” gives identity of clustered OTUs for comparison between categories.

| | Phylum | Class | Order | Family | Genus | OTU-ID name |
|---|---|---|---|---|---|---|
| 1 | Proteobacteria | Betaproteobacteria | Burkholderiales | Comamonadaceae | NA | 1 |
| 2 | Proteobacteria | Alphaproteobacteria | Rhizobiales | Bradyrhizobiaceae | Bradyrhizobium | 2 |
| 3 | Proteobacteria | Alphaproteobacteria | Caulobacterales | Caulobacteraceae | Caulobacter | 3 |
| 4 | Proteobacteria | Alphaproteobacteria | Rhizobiales | NA | NA | 9 |
| 5 | Proteobacteria | Alphaproteobacteria | Sphingomonadales | Sphingomonadaceae | Sphingomonas | 15 |
| 6 | Proteobacteria | Alphaproteobacteria | Rhizobiales | Rhizobiaceae | Agrobacterium | 16 |
| 7 | Proteobacteria | Alphaproteobacteria | Rhizobiales | Methylobacteriaceae | NA | 20 |
| 8 | Actinobacteria | Actinobacteria | Actinomycetales | Microbacteriaceae | NA | 75 |
| 1 | Proteobacteria | Betaproteobacteria | Burkholderiales | Comamonadaceae | NA | 1 |
| 2 | Proteobacteria | Alphaproteobacteria | Rhizobiales | Bradyrhizobiaceae | Bradyrhizobium | 2 |
| 3 | Proteobacteria | Alphaproteobacteria | Caulobacterales | Caulobacteraceae | Caulobacter | 3 |
| 4 | Proteobacteria | Alphaproteobacteria | Sphingomonadales | Sphingomonadaceae | Novosphingobium | 4 |
| 5 | Proteobacteria | Alphaproteobacteria | Rhizobiales | NA | NA | 9 |
| 6 | Proteobacteria | Alphaproteobacteria | Sphingomonadales | Sphingomonadaceae | Sphingomonas | 15 |
| 7 | Proteobacteria | Alphaproteobacteria | Rhizobiales | Rhizobiaceae | Agrobacterium | 16 |
| 8 | Proteobacteria | Alphaproteobacteria | Rhizobiales | Methylobacteriaceae | NA | 20 |
| 9 | Proteobacteria | Alphaproteobacteria | Rhodobacterales | Hyphomonadaceae | NA | 45 |
| 10 | Proteobacteria | Alphaproteobacteria | Sphingomonadales | Sphingomonadaceae | Sphingomonas | 57 |
| 11 | Actinobacteria | Actinobacteria | Actinomycetales | Microbacteriaceae | NA | 75 |
| 12 | Proteobacteria | Alphaproteobacteria | Rhizobiales | Methylobacteriaceae | Methylobacterium | 289 |
| 13 | Proteobacteria | Alphaproteobacteria | Caulobacterales | Caulobacteraceae | NA | 25548 |
| 1 | Proteobacteria | Betaproteobacteria | Burkholderiales | Comamonadaceae | NA | 1 |
| 2 | Proteobacteria | Alphaproteobacteria | Rhizobiales | Bradyrhizobiaceae | Bradyrhizobium | 2 |
| 3 | Proteobacteria | Alphaproteobacteria | Caulobacterales | Caulobacteraceae | Caulobacter | 3 |
| 4 | Proteobacteria | Alphaproteobacteria | Rhodospirillales | Rhodospirillaceae | NA | 8 |
| 5 | Proteobacteria | Alphaproteobacteria | Rhizobiales | NA | NA | 9 |
| 6 | Proteobacteria | Gammaproteobacteria | Pseudomonadales | Moraxellaceae | Enhydrobacter | 13 |
| 7 | Proteobacteria | Alphaproteobacteria | Sphingomonadales | Sphingomonadaceae | Sphingomonas | 15 |

| | | | | | | |
|---|---|---|---|---|---|---|
| 8 | Proteobacteria | Alphaproteobacteria | Rhizobiales | Rhizobiaceae | Agrobacterium | 16 |
| 9 | Proteobacteria | Alphaproteobacteria | Rhizobiales | Methylobacteriaceae | NA | 20 |
| 10 | Proteobacteria | Alphaproteobacteria | Caulobacterales | Caulobacteraceae | Phenylobacterium | 33 |
| 11 | Bacteroidetes | Flavobacteriia | Flavobacteriales | Flavobacteriaceae | Flavobacterium | 37 |
| 12 | Proteobacteria | Alphaproteobacteria | Caulobacterales | Caulobacteraceae | NA | 41 |
| 13 | Proteobacteria | Betaproteobacteria | Burkholderiales | Comamonadaceae | Hylemonella | 55 |
| 14 | Proteobacteria | Gammaproteobacteria | Pseudomonadales | Pseudomonadaceae | Pseudomonas | 60 |
| 15 | Actinobacteria | Actinobacteria | Actinomycetales | Brevibacteriaceae | Brevibacterium | 62 |
| 16 | Actinobacteria | Actinobacteria | Actinomycetales | Microbacteriaceae | NA | 75 |
| 17 | Planctomycetes | Phycisphaerae | Phycisphaerales | NA | NA | 84 |
| 18 | TM7 | TM7-3 | NA | NA | NA | 85 |
| 19 | Proteobacteria | Gammaproteobacteria | Pseudomonadales | Moraxellaceae | Acinetobacter | 111 |
| 20 | Proteobacteria | Alphaproteobacteria | Rhizobiales | Methylocystaceae | Methylopila | 119 |
| 21 | Bacteroidetes | Flavobacteriia | Flavobacteriales | [Weeksellaceae] | Chryseobacterium | 126 |
| 22 | Proteobacteria | Alphaproteobacteria | Sphingomonadales | Sphingomonadaceae | Sphingobium | 150 |
| 23 | Proteobacteria | Deltaproteobacteria | Bdellovibrionales | Bdellovibrionaceae | Bdellovibrio | 171 |
| 24 | Actinobacteria | Actinobacteria | Actinomycetales | Williamsiaceae | Williamsia | 191 |
| 25 | Proteobacteria | Alphaproteobacteria | Caulobacterales | Caulobacteraceae | NA | 254 |
| 26 | Actinobacteria | Actinobacteria | Actinomycetales | Nocardiaceae | Rhodococcus | 317 |
| 27 | Proteobacteria | Gammaproteobacteria | Pseudomonadales | Pseudomonadaceae | Pseudomonas | 338 |
| 28 | Proteobacteria | Alphaproteobacteria | Rhodospirillales | Acetobacteraceae | NA | 376 |
| 29 | Armatimonadetes | [Fimbriimonadia] | [Fimbriimonadales] | [Fimbriimonadaceae] | Fimbriimonas | 403 |
| 30 | Bacteroidetes | Flavobacteriia | Flavobacteriales | [Weeksellaceae] | Chryseobacterium | 448 |
| 31 | Proteobacteria | Alphaproteobacteria | Rhizobiales | Brucellaceae | Ochrobactrum | 718 |
| 32 | WPS-2 | NA | NA | NA | NA | 1003 |
| 33 | Proteobacteria | Alphaproteobacteria | Rhizobiales | Rhizobiaceae | Agrobacterium | 2702 |
| 34 | Proteobacteria | Betaproteobacteria | Burkholderiales | Comamonadaceae | NA | 9064 |
| 35 | Proteobacteria | Alphaproteobacteria | Rhizobiales | Xanthobacteraceae | Xanthobacter | 9623 |
| 36 | Actinobacteria | Actinobacteria | Actinomycetales | Microbacteriaceae | NA | 15174 |
| 37 | Proteobacteria | Alphaproteobacteria | Rhizobiales | NA | NA | 24425 |
| 38 | Proteobacteria | Alphaproteobacteria | Caulobacterales | Caulobacteraceae | Brevundimonas | 24749 |
| 39 | Proteobacteria | Alphaproteobacteria | Rhodobacterales | Rhodobacteraceae | NA | 25533 |
| 40 | Proteobacteria | Alphaproteobacteria | Rhizobiales | NA | NA | 25657 |
| 41 | Proteobacteria | Alphaproteobacteria | Caulobacterales | Caulobacteraceae | NA | 27237 |
| 42 | Proteobacteria | Alphaproteobacteria | Caulobacterales | Caulobacteraceae | NA | 27622 |
| 43 | Proteobacteria | Alphaproteobacteria | Caulobacterales | Caulobacteraceae | NA | 33484 |
| 44 | Proteobacteria | Alphaproteobacteria | Caulobacterales | Caulobacteraceae | NA | 34670 |
| 45 | Proteobacteria | Alphaproteobacteria | Caulobacterales | Caulobacteraceae | NA | 36313 |

| | | | | | | |
|---|---|---|---|---|---|---|
| 46 | Proteobacteria | Alphaproteobacteria | Caulobacterales | Caulobacteraceae | NA | 36581 |
| 47 | Proteobacteria | Alphaproteobacteria | Caulobacterales | Caulobacteraceae | NA | 39507 |
| 1 | Proteobacteria | Betaproteobacteria | Burkholderiales | Comamonadaceae | NA | 1 |
| 2 | Proteobacteria | Alphaproteobacteria | Rhizobiales | Bradyrhizobiaceae | Bradyrhizobium | 2 |
| 3 | Proteobacteria | Alphaproteobacteria | Caulobacterales | Caulobacteraceae | Caulobacter | 3 |
| 4 | Proteobacteria | Alphaproteobacteria | Sphingomonadales | Sphingomonadaceae | Novosphingobium | 4 |
| 5 | Proteobacteria | Betaproteobacteria | Burkholderiales | Comamonadaceae | Comamonas | 5 |
| 6 | Proteobacteria | Alphaproteobacteria | Rhodospirillales | Rhodospirillaceae | NA | 8 |
| 7 | Proteobacteria | Alphaproteobacteria | Rhizobiales | NA | NA | 9 |
| 8 | Proteobacteria | Gammaproteobacteria | Pseudomonadales | Moraxellaceae | Enhydrobacter | 13 |
| 9 | Proteobacteria | Alphaproteobacteria | Sphingomonadales | Sphingomonadaceae | Sphingomonas | 15 |
| 10 | Proteobacteria | Alphaproteobacteria | Rhizobiales | Rhizobiaceae | Agrobacterium | 16 |
| 11 | Proteobacteria | Alphaproteobacteria | Rhizobiales | Methylobacteriaceae | NA | 20 |
| 12 | Proteobacteria | Alphaproteobacteria | Caulobacterales | Caulobacteraceae | Phenylobacterium | 33 |
| 13 | Bacteroidetes | Flavobacteriia | Flavobacteriales | Flavobacteriaceae | Flavobacterium | 37 |
| 14 | Proteobacteria | Alphaproteobacteria | Caulobacterales | Caulobacteraceae | NA | 41 |
| 15 | Proteobacteria | Betaproteobacteria | Burkholderiales | Comamonadaceae | Hylemonella | 55 |
| 16 | Proteobacteria | Alphaproteobacteria | Sphingomonadales | Sphingomonadaceae | Sphingomonas | 57 |
| 17 | Proteobacteria | Gammaproteobacteria | Pseudomonadales | Pseudomonadaceae | Pseudomonas | 60 |
| 18 | Actinobacteria | Actinobacteria | Actinomycetales | Brevibacteriaceae | Brevibacterium | 62 |
| 19 | Bacteroidetes | Sphingobacteriia | Sphingobacteriales | NA | NA | 68 |
| 20 | Actinobacteria | Actinobacteria | Actinomycetales | Microbacteriaceae | NA | 75 |
| 21 | Planctomycetes | Phycisphaerae | Phycisphaerales | NA | NA | 84 |
| 22 | TM7 | TM7-3 | NA | NA | NA | 85 |
| 23 | Proteobacteria | Gammaproteobacteria | Pseudomonadales | Moraxellaceae | Acinetobacter | 111 |
| 24 | Proteobacteria | Alphaproteobacteria | Rhizobiales | Methylocystaceae | Methylopila | 119 |
| 25 | Bacteroidetes | Flavobacteriia | Flavobacteriales | [Weeksellaceae] | Chryseobacterium | 126 |
| 26 | WPS-2 | NA | NA | NA | NA | 146 |
| 27 | Proteobacteria | Alphaproteobacteria | Sphingomonadales | Sphingomonadaceae | Sphingobium | 150 |
| 28 | Proteobacteria | Deltaproteobacteria | Bdellovibrionales | Bdellovibrionaceae | Bdellovibrio | 171 |
| 29 | Actinobacteria | Actinobacteria | Actinomycetales | Williamsiaceae | Williamsia | 191 |
| 30 | Planctomycetes | Planctomycetia | Planctomycetales | Planctomycetaceae | Planctomyces | 226 |
| 31 | Proteobacteria | Alphaproteobacteria | Rhizobiales | Methylobacteriaceae | NA | 233 |
| 32 | Proteobacteria | Alphaproteobacteria | Caulobacterales | Caulobacteraceae | NA | 254 |
| 33 | Proteobacteria | Alphaproteobacteria | Rhizobiales | Methylobacteriaceae | Methylobacterium | 289 |
| 34 | Bacteroidetes | Sphingobacteriia | Sphingobacteriales | Sphingobacteriaceae | Pedobacter | 292 |
| 35 | Actinobacteria | Actinobacteria | Actinomycetales | Nocardiaceae | Rhodococcus | 317 |
| 36 | Proteobacteria | Gammaproteobacte | Pseudomonadal | Pseudomonadaceae | Pseudomonas | 338 |

| | | | | | | |
|---|---|---|---|---|---|---|
| | | ria | es | | | |
| 37 | Proteobacteria | Alphaproteobacteria | Rhodospirillales | Acetobacteraceae | NA | 376 |
| 38 | Bacteroidetes | Cytophagia | Cytophagales | Cytophagaceae | Spirosoma | 381 |
| 39 | Armatimonadetes | [Fimbriimonadia] | [Fimbriimonadales] | [Fimbriimonadaceae] | Fimbriimonas | 403 |
| 40 | Firmicutes | Bacilli | Bacillales | Planococcaceae | Lysinibacillus | 447 |
| 41 | Bacteroidetes | Flavobacteriia | Flavobacteriales | [Weeksellaceae] | Chryseobacterium | 448 |
| 42 | Proteobacteria | Alphaproteobacteria | Rhodospirillales | Rhodospirillaceae | Azospirillum | 533 |
| 43 | Proteobacteria | Alphaproteobacteria | Rhizobiales | Brucellaceae | Ochrobactrum | 718 |
| 44 | WPS-2 | NA | NA | NA | NA | 1003 |
| 45 | Actinobacteria | Actinobacteria | Actinomycetales | Micrococcaceae | Citricoccus | 1261 |
| 46 | Proteobacteria | Alphaproteobacteria | Rhizobiales | Rhizobiaceae | Agrobacterium | 2702 |
| 47 | Proteobacteria | Deltaproteobacteria | Myxococcales | 0319-6G20 | NA | 3053 |
| 48 | Proteobacteria | Betaproteobacteria | Burkholderiales | Comamonadaceae | NA | 9064 |
| 49 | Proteobacteria | Alphaproteobacteria | Rhizobiales | Xanthobacteraceae | Xanthobacter | 9623 |
| 50 | Firmicutes | Bacilli | Lactobacillales | Streptococcaceae | Streptococcus | 11155 |
| 51 | Proteobacteria | Alphaproteobacteria | Rhizobiales | NA | NA | 14526 |
| 52 | Actinobacteria | Actinobacteria | Actinomycetales | Microbacteriaceae | NA | 15174 |
| 53 | Proteobacteria | Betaproteobacteria | Burkholderiales | Comamonadaceae | NA | 19848 |
| 54 | Proteobacteria | Gammaproteobacteria | Pseudomonadales | Pseudomonadaceae | Pseudomonas | 21114 |
| 55 | Proteobacteria | Alphaproteobacteria | Sphingomonadales | Sphingomonadaceae | NA | 21892 |
| 56 | Proteobacteria | Alphaproteobacteria | Rhizobiales | NA | NA | 24425 |
| 57 | Proteobacteria | Alphaproteobacteria | Caulobacterales | Caulobacteraceae | Brevundimonas | 24749 |
| 58 | Proteobacteria | Alphaproteobacteria | Rhizobiales | Methylobacteriaceae | NA | 25305 |
| 59 | Proteobacteria | Alphaproteobacteria | Rhodobacterales | Rhodobacteraceae | NA | 25533 |
| 60 | Proteobacteria | Alphaproteobacteria | Rhizobiales | NA | NA | 25657 |
| 61 | Proteobacteria | Alphaproteobacteria | Caulobacterales | Caulobacteraceae | NA | 27237 |
| 62 | Proteobacteria | Alphaproteobacteria | Caulobacterales | Caulobacteraceae | NA | 27622 |
| 63 | Proteobacteria | Alphaproteobacteria | Caulobacterales | Caulobacteraceae | NA | 28140 |
| 64 | Proteobacteria | Alphaproteobacteria | Caulobacterales | Caulobacteraceae | NA | 33484 |
| 65 | Proteobacteria | Alphaproteobacteria | Caulobacterales | Caulobacteraceae | NA | 34670 |
| 66 | Proteobacteria | Alphaproteobacteria | Rhizobiales | NA | NA | 35651 |
| 67 | Proteobacteria | Alphaproteobacteria | Caulobacterales | Caulobacteraceae | NA | 36313 |
| 68 | Proteobacteria | Alphaproteobacteria | Caulobacterales | Caulobacteraceae | NA | 36581 |
| 69 | Proteobacteria | Alphaproteobacteria | Rhizobiales | Rhizobiaceae | Agrobacterium | 39228 |
| 70 | Proteobacteria | Alphaproteobacteria | Caulobacterales | Caulobacteraceae | NA | 39507 |
| 71 | Proteobacteria | Alphaproteobacteria | Caulobacterales | Caulobacteraceae | NA | 41388 |
| 72 | Proteobacteria | Alphaproteobacteria | Caulobacterales | Caulobacteraceae | NA | 44242 |

| | | | | | | |
|---|---|---|---|---|---|---|
| 1 | Proteobacteria | Betaproteobacteria | Burkholderiales | Comamonadaceae | NA | 1 |
| 2 | Proteobacteria | Alphaproteobacteri a | Rhizobiales | Bradyrhizobiaceae | Bradyrhizobium | 2 |
| 3 | Proteobacteria | Alphaproteobacteri a | Caulobacterales | Caulobacteraceae | Caulobacter | 3 |
| 4 | Proteobacteria | Alphaproteobacteri a | Rhodospirillales | Rhodospirillaceae | NA | 8 |
| 5 | Proteobacteria | Alphaproteobacteri a | Rhizobiales | NA | NA | 9 |
| 6 | Proteobacteria | Gammaproteobacte ria | Pseudomonadal es | Moraxellaceae | Enhydrobacter | 13 |
| 7 | TM7 | TM7-3 | EW055 | NA | NA | 14 |
| 8 | Proteobacteria | Alphaproteobacteri a | Sphingomonadal es | Sphingomonadacea e | Sphingomonas | 15 |
| 9 | Proteobacteria | Alphaproteobacteri a | Rhizobiales | Rhizobiaceae | Agrobacterium | 16 |
| 10 | Proteobacteria | Alphaproteobacteri a | Rhizobiales | Methylobacteriacea e | NA | 20 |
| 11 | Proteobacteria | Alphaproteobacteri a | Caulobacterales | Caulobacteraceae | Phenylobacterium | 33 |
| 12 | Proteobacteria | Gammaproteobacte ria | Xanthomonadale s | Xanthomonadaceae | Pseudoxanthomo nas | 34 |
| 13 | Bacteroidetes | Flavobacteriia | Flavobacteriales | Flavobacteriaceae | Flavobacterium | 37 |
| 14 | Actinobacteria | Actinobacteria | Actinomycetales | Mycobacteriaceae | Mycobacterium | 38 |
| 15 | Proteobacteria | Alphaproteobacteri a | Caulobacterales | Caulobacteraceae | NA | 41 |
| 16 | Proteobacteria | Betaproteobacteria | Burkholderiales | Comamonadaceae | Hylemonella | 55 |
| 17 | Proteobacteria | Gammaproteobacte ria | Pseudomonadal es | Pseudomonadaceae | Pseudomonas | 60 |
| 18 | Actinobacteria | Actinobacteria | Actinomycetales | Brevibacteriaceae | Brevibacterium | 62 |
| 19 | Actinobacteria | Actinobacteria | Actinomycetales | Microbacteriaceae | NA | 75 |
| 20 | Bacteroidetes | Flavobacteriia | Flavobacteriales | [Weeksellaceae] | Chryseobacterium | 80 |
| 21 | Planctomycete s | Phycisphaerae | Phycisphaerales | NA | NA | 84 |
| 22 | TM7 | TM7-3 | NA | NA | NA | 85 |
| 23 | Proteobacteria | Gammaproteobacte ria | Pseudomonadal es | Moraxellaceae | Acinetobacter | 111 |
| 24 | Proteobacteria | Gammaproteobacte ria | Legionellales | Legionellaceae | NA | 117 |
| 25 | Proteobacteria | Alphaproteobacteri a | Rhizobiales | Methylocystaceae | Methylopila | 119 |
| 26 | Bacteroidetes | Flavobacteriia | Flavobacteriales | [Weeksellaceae] | Chryseobacterium | 126 |
| 27 | Proteobacteria | Alphaproteobacteri a | Sphingomonadal es | Sphingomonadacea e | Sphingobium | 150 |
| 28 | Proteobacteria | Deltaproteobacteria | Bdellovibrionales | Bdellovibrionaceae | Bdellovibrio | 171 |
| 29 | Verrucomicrobi a | Verrucomicrobiae | Verrucomicrobial es | Verrucomicrobiacea e | Prosthecobacter | 180 |
| 30 | Actinobacteria | Actinobacteria | Actinomycetales | Williamsiaceae | Williamsia | 191 |
| 31 | Proteobacteria | Alphaproteobacteri a | BD7-3 | NA | NA | 198 |
| 32 | Proteobacteria | Alphaproteobacteri a | Rhodobacterales | Rhodobacteraceae | Rhodobacter | 200 |
| 33 | Proteobacteria | Alphaproteobacteri a | Caulobacterales | Caulobacteraceae | NA | 254 |
| 34 | Proteobacteria | Gammaproteobacte ria | Pseudomonadal es | Moraxellaceae | Acinetobacter | 258 |
| 35 | Proteobacteria | Gammaproteobacte ria | Xanthomonadale s | Xanthomonadaceae | Dokdonella | 287 |
| 36 | Actinobacteria | Actinobacteria | Actinomycetales | Nocardiaceae | Rhodococcus | 317 |
| 37 | Bacteroidetes | Sphingobacteriia | Sphingobacterial es | Sphingobacteriacea e | Pedobacter | 318 |
| 38 | Proteobacteria | Gammaproteobacte ria | Pseudomonadal es | Pseudomonadaceae | Pseudomonas | 338 |

| | | | | | | |
|---|---|---|---|---|---|---|
| 39 | Proteobacteria | Gammaproteobacteria | Legionellales | Coxiellaceae | Aquicella | 367 |
| 40 | Proteobacteria | Alphaproteobacteria | Rhodospirillales | Acetobacteraceae | NA | 376 |
| 41 | Armatimonadetes | [Fimbriimonadia] | [Fimbriimonadales] | [Fimbriimonadaceae] | Fimbriimonas | 403 |
| 42 | Bacteroidetes | Flavobacteriia | Flavobacteriales | [Weeksellaceae] | Chryseobacterium | 448 |
| 43 | Proteobacteria | Alphaproteobacteria | Rhizobiales | Beijerinckiaceae | NA | 559 |
| 44 | Proteobacteria | Alphaproteobacteria | Rhizobiales | Brucellaceae | Ochrobactrum | 718 |
| 45 | Proteobacteria | Gammaproteobacteria | Pseudomonadales | Moraxellaceae | Acinetobacter | 820 |
| 46 | Proteobacteria | Deltaproteobacteria | Bdellovibrionales | Bdellovibrionaceae | Bdellovibrio | 910 |
| 47 | Proteobacteria | Gammaproteobacteria | Xanthomonadales | Xanthomonadaceae | NA | 959 |
| 48 | WPS-2 | NA | NA | NA | NA | 1003 |
| 49 | Bacteroidetes | Flavobacteriia | Flavobacteriales | [Weeksellaceae] | Chryseobacterium | 1090 |
| 50 | Bacteroidetes | [Saprospirae] | [Saprospirales] | Chitinophagaceae | NA | 1186 |
| 51 | Planctomycetes | Planctomycetia | Gemmatales | Isosphaeraceae | NA | 1648 |
| 52 | Proteobacteria | Alphaproteobacteria | Rhizobiales | Rhizobiaceae | Agrobacterium | 2702 |
| 53 | Actinobacteria | Actinobacteria | Actinomycetales | Gordoniaceae | NA | 4496 |
| 54 | Proteobacteria | Betaproteobacteria | Burkholderiales | Comamonadaceae | Delftia | 4964 |
| 55 | Proteobacteria | Alphaproteobacteria | Caulobacterales | Caulobacteraceae | NA | 6141 |
| 56 | Proteobacteria | Betaproteobacteria | Burkholderiales | Comamonadaceae | NA | 9064 |
| 57 | Proteobacteria | Alphaproteobacteria | Rhizobiales | Xanthobacteraceae | Xanthobacter | 9623 |
| 58 | Proteobacteria | Betaproteobacteria | Burkholderiales | Comamonadaceae | NA | 13280 |
| 59 | Proteobacteria | Alphaproteobacteria | Rhizobiales | Xanthobacteraceae | NA | 13697 |
| 60 | Actinobacteria | Actinobacteria | Actinomycetales | Microbacteriaceae | NA | 15174 |
| 61 | Proteobacteria | Betaproteobacteria | Burkholderiales | Comamonadaceae | NA | 16770 |
| 62 | Actinobacteria | Actinobacteria | Actinomycetales | Mycobacteriaceae | Mycobacterium | 18398 |
| 63 | Actinobacteria | Actinobacteria | Actinomycetales | Mycobacteriaceae | Mycobacterium | 18530 |
| 64 | Actinobacteria | Actinobacteria | Actinomycetales | Mycobacteriaceae | Mycobacterium | 20814 |
| 65 | Proteobacteria | Betaproteobacteria | Burkholderiales | Comamonadaceae | Roseateles | 21094 |
| 66 | Proteobacteria | Betaproteobacteria | Burkholderiales | Comamonadaceae | Roseateles | 21116 |
| 67 | Proteobacteria | Betaproteobacteria | Burkholderiales | Comamonadaceae | Roseateles | 22107 |
| 68 | Proteobacteria | Betaproteobacteria | Burkholderiales | Comamonadaceae | Roseateles | 22334 |
| 69 | Proteobacteria | Alphaproteobacteria | Rhizobiales | NA | NA | 24425 |
| 70 | Proteobacteria | Alphaproteobacteria | Caulobacterales | Caulobacteraceae | Brevundimonas | 24749 |
| 71 | Proteobacteria | Alphaproteobacteria | Rhizobiales | NA | NA | 25154 |
| 72 | Proteobacteria | Alphaproteobacteria | Caulobacterales | Caulobacteraceae | NA | 25286 |
| 73 | Proteobacteria | Alphaproteobacteria | Sphingomonadales | Sphingomonadaceae | NA | 25380 |
| 74 | Proteobacteria | Alphaproteobacteria | Caulobacterales | Caulobacteraceae | NA | 25387 |
| 75 | Proteobacteria | Alphaproteobacteria | Rhodobacterales | Rhodobacteraceae | NA | 25533 |
| 76 | Proteobacteria | Alphaproteobacteria | Caulobacterales | Caulobacteraceae | NA | 25548 |
| 77 | Proteobacteria | Alphaproteobacteria | Caulobacterales | Caulobacteraceae | NA | 25552 |

| | | | | | | |
|---|---|---|---|---|---|---|
| 78 | Proteobacteria | Alphaproteobacteria | Caulobacterales | Caulobacteraceae | NA | 25649 |
| 79 | Proteobacteria | Alphaproteobacteria | Caulobacterales | Caulobacteraceae | NA | 25650 |
| 80 | Proteobacteria | Alphaproteobacteria | Rhizobiales | NA | NA | 25657 |
| 81 | Proteobacteria | Alphaproteobacteria | Caulobacterales | Caulobacteraceae | NA | 27237 |
| 82 | Proteobacteria | Alphaproteobacteria | Caulobacterales | Caulobacteraceae | NA | 27622 |
| 83 | Proteobacteria | Betaproteobacteria | Burkholderiales | Comamonadaceae | Roseateles | 27910 |
| 84 | Proteobacteria | Alphaproteobacteria | Caulobacterales | Caulobacteraceae | NA | 27981 |
| 85 | Proteobacteria | Alphaproteobacteria | Caulobacterales | Caulobacteraceae | NA | 28085 |
| 86 | Proteobacteria | Alphaproteobacteria | Caulobacterales | Caulobacteraceae | NA | 28098 |
| 87 | Proteobacteria | Alphaproteobacteria | Caulobacterales | Caulobacteraceae | NA | 28102 |
| 88 | Proteobacteria | Alphaproteobacteria | Caulobacterales | Caulobacteraceae | NA | 28130 |
| 89 | Proteobacteria | Alphaproteobacteria | Caulobacterales | Caulobacteraceae | Phenylobacterium | 32272 |
| 90 | Proteobacteria | Alphaproteobacteria | Caulobacterales | Caulobacteraceae | Phenylobacterium | 32696 |
| 91 | Proteobacteria | Alphaproteobacteria | Caulobacterales | Caulobacteraceae | NA | 33213 |
| 92 | Proteobacteria | Alphaproteobacteria | Caulobacterales | Caulobacteraceae | Phenylobacterium | 33240 |
| 93 | Proteobacteria | Alphaproteobacteria | Caulobacterales | Caulobacteraceae | NA | 33281 |
| 94 | Proteobacteria | Alphaproteobacteria | Caulobacterales | Caulobacteraceae | NA | 33484 |
| 95 | Proteobacteria | Alphaproteobacteria | Caulobacterales | Caulobacteraceae | NA | 33496 |
| 96 | Proteobacteria | Alphaproteobacteria | Caulobacterales | Caulobacteraceae | NA | 33868 |
| 97 | Proteobacteria | Alphaproteobacteria | Caulobacterales | Caulobacteraceae | Mycoplana | 33931 |
| 98 | Proteobacteria | Alphaproteobacteria | Caulobacterales | Caulobacteraceae | NA | 34122 |
| 99 | Proteobacteria | Alphaproteobacteria | Caulobacterales | Caulobacteraceae | NA | 34125 |
| 100 | Proteobacteria | Alphaproteobacteria | Caulobacterales | Caulobacteraceae | NA | 34670 |
| 101 | Proteobacteria | Alphaproteobacteria | Caulobacterales | Caulobacteraceae | NA | 36313 |
| 102 | Proteobacteria | Alphaproteobacteria | Caulobacterales | Caulobacteraceae | NA | 36569 |
| 103 | Proteobacteria | Alphaproteobacteria | Caulobacterales | Caulobacteraceae | NA | 36581 |
| 104 | Proteobacteria | Alphaproteobacteria | Caulobacterales | Caulobacteraceae | NA | 36622 |
| 105 | Proteobacteria | Alphaproteobacteria | Caulobacterales | Caulobacteraceae | NA | 38225 |
| 106 | Proteobacteria | Alphaproteobacteria | Caulobacterales | Caulobacteraceae | NA | 39507 |
| 107 | Proteobacteria | Alphaproteobacteria | Rhodospirillales | Rhodospirillaceae | NA | 43316 |

**Table S3:** 'Household-specific core communities' in real bath toys originated from five different Swiss households, with bath toys provided ranging between 1 – 6 bath toys per household. The OTUs in each core community are (1) present in all samples from the household, and (2) not present in other households; while shared OTUs are (1) present in all samples from the household, but (2) not necessarily exclusively.

| | n(bath toys) / household | Sum of reads | n(household specific core OTUs) | % of core-reads / total number of reads within household | n(shared OTUs) / household |
|---|---|---|---|---|---|
| **Household_I** | 1 | 30'133 | 33 | 0.1 | - |
| **Household_II** | 2 | 60'266 | 1'289 | 2.9 | 1'445 |
| **Household_III** | 3 | 90'399 | 741 | 2.1 | 124 |
| **Household_IV** | 5 | 150'665 | 1'059 | 1.1 | 41 |
| **Household_V** | 6 | 180'798 | 3'904 | 4.2 | 29 |

**Table S4:** Number of operational taxonomic units (OTUs) per individual bath toy and Shannon-Wiener index (H') as a measure of diversity.

| Bath toy | Number of observed OTUs | H' |
|---|---|---|
| Real bath toy 01 | 2'826 | 0.52 |
| Real bath toy 02 | 239 | 0.31 |
| Real bath toy 03 | 597 | 0.43 |
| Real bath toy 04 | 3'199 | 0.62 |
| Real bath toy 05 | 3'483 | 0.52 |
| Real bath toy 06 | 2'458 | 0.47 |
| Real bath toy 07 | 281 | 0.35 |
| Real bath toy 08 | 192 | 0.27 |
| Real bath toy 09 | 6'196 | 0.71 |
| Real bath toy 10 | 3'562 | 0.57 |
| Real bath toy 11 | 429 | 0.40 |
| Real bath toy 12 | 204 | 0.26 |
| Real bath toy 13 | 329 | 0.39 |
| Real bath toy 14 | 311 | 0.33 |
| Real bath toy 15 | No data available | |
| Real bath toy 16 | 335 | 0.33 |
| Real bath toy 17 | 527 | 0.35 |
| Real bath toy 18 | No data available | |
| Real bath toy 19 | 435 | 0.32 |
| Clean water control | 214 | 0.26 |
| Clean water control | 188 | 0.23 |
| Clean water control | 265 | 0.17 |
| Dirty water control | 269 | 0.32 |
| Dirty water control | 268 | 0.25 |
| Dirty water control | 252 | 0.25 |

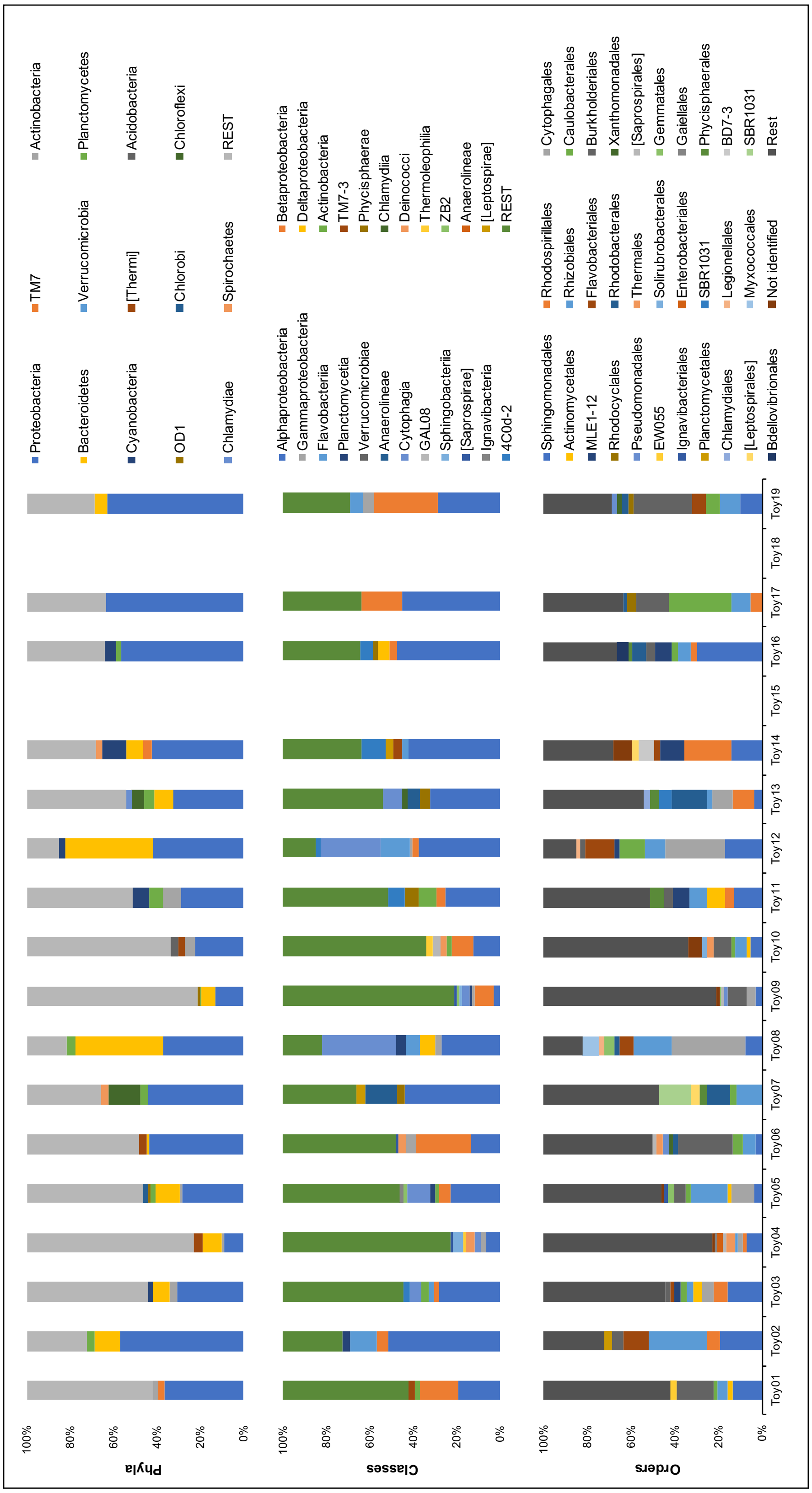


**Figure S5:** Classification of abundant OTUs in real bath toy biofilms. The ten most abundant OTUs for each single bath toy (i.e., OTUs with the most numbers of reads with all samples set to an even depth of 30’133 reads), originating from random households, were separated on different classification levels (phylum, class, order) to highlight differences in community compositions.

**Table S5:** Comparison of most abundant operational taxonomic units (OTUs) in control bath toy biofilms. Most abundant OTUs in clean and dirty water control biofilms are specified on different classification levels. Bold highlighted OTUs were dominant in both clean and dirty water controls.

| | | Phylum | Class | Order | Family | Genus |
|---|---|---|---|---|---|---|
| Clean water controls | | **Proteobacteria** | **Alphaproteobacteria** | **Caulobacterales** | **Caulobacteraceae** | **Caulobacter** |
| | | **Proteobacteria** | **Alphaproteobacteria** | **Rhodospirillales** | **Rhodospirillaceae** | **NA** |
| | | **Proteobacteria** | **Alphaproteobacteria** | **Caulobacterales** | **Caulobacteraceae** | **NA** |
| | | Proteobacteria | Betaproteobacteria | Burkholderiales | Comamonadaceae | NA |
| | | Proteobacteria | Alphaproteobacteria | Sphingomonadales | Sphingomonadaceae | Sphingomonas |
| | | Proteobacteria | Alphaproteobacteria | Rhizobiales | Methylobacteriaceae | NA |
| | | Proteobacteria | Alphaproteobacteria | Caulobacterales | Caulobacteraceae | NA |
| | | Proteobacteria | Alphaproteobacteria | Sphingomonadales | Sphingomonadaceae | Sphingomonas |
| | | Planctomycetes | Phycisphaerae | Phycisphaerales | NA | NA |
| | | Proteobacteria | Betaproteobacteria | Burkholderiales | Comamonadaceae | NA |
| | | Rest | / | / | / | / |
| | | | | | | |
| | | Phylum | Class | Order | Family | Genus |
| Dirty water controls | | **Proteobacteria** | **Alphaproteobacteria** | **Caulobacterales** | **Caulobacteraceae** | **Caulobacter** |
| | | **Proteobacteria** | **Alphaproteobacteria** | **Rhodospirillales** | **Rhodospirillaceae** | **NA** |
| | | **Proteobacteria** | **Alphaproteobacteria** | **Caulobacterales** | **Caulobacteraceae** | **NA** |
| | | Proteobacteria | Alphaproteobacteria | Rhizobiales | Rhizobiaceae | Agrobacterium |
| | | Proteobacteria | Alphaproteobacteria | Caulobacterales | Caulobacteraceae | Phenylobacterium |
| | | Actinobacteria | Actinobacteria | Actinomycetales | Mycobacteraceae | Mycobacterium |
| | | Bacteroidetes | Flavobacteriia | Flavobacteriales | [[Weeksellaceae] | Chryseobacterium |
| | | TM7 | TM/-3 | NA | NA | NA |
| | | Proteobacteria | Betaproteobacteria | Burkholderiales | Comamonadaceae | NA |
| | | Proteobacteria | Alphaproteobacteria | Caulobacterales | Caulobacteraceae | NA |
| | | Rest | / | / | / | / |

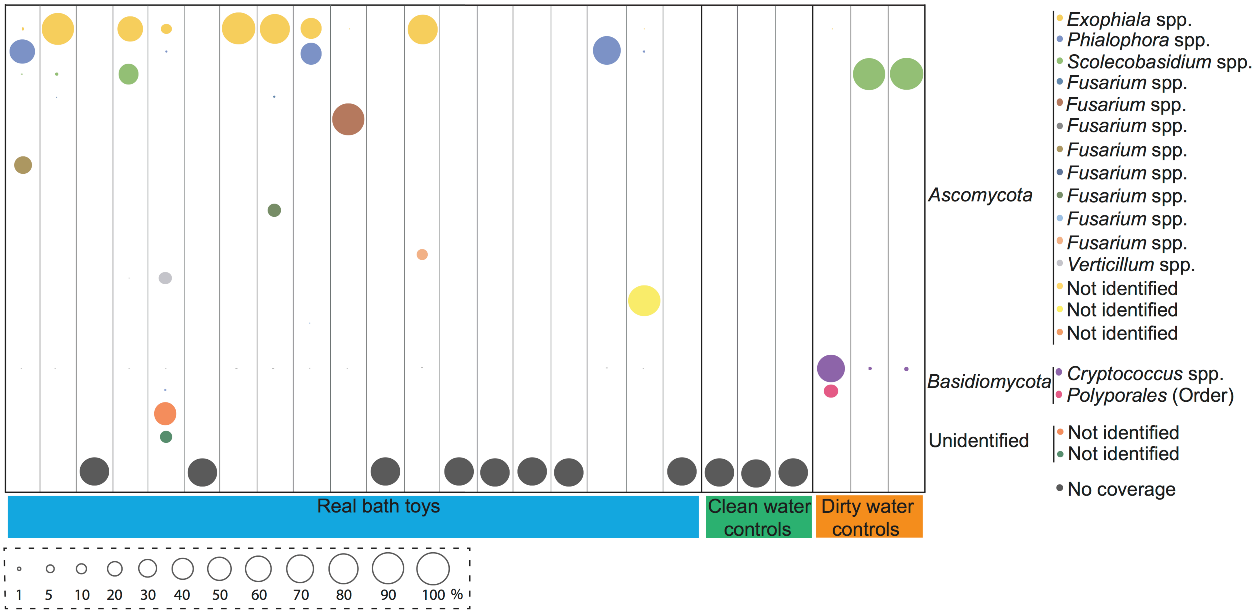


**Figure S6:** Dominant operational taxonomic units (OTUs) in fungal communities of bath toy biofilms. Colors represent different OTUs on genus level, with the majority belonging to the phylum Ascomycota and the rest either to Basidiomycota or being unidentified. Samples indicated with black circles did not show any results for fungi representatives. In general, the size of the circles indicates the absolute abundance of each OTU, with bigger volumes indicating higher percentages.

**Table S6:** Conventional plating results testing biofilms from the inner surface of bath toys for several indicator bacteria or groups. Samples were taken from either real bath toys (A) which were used randomly in different households or from control bath toys (B) which were used either with clean water prior to bathing (clean water controls) or with used water after bathing (dirty water controls). Compact Dry Plates were used for testing the presence of *Pseudomonas aeruginosa*, *Listeria* spp., *Enterococci* spp., Coliforms, and *Escherichia coli*. Special agar plates (Legionella BMP α Selective Medium) were used for the detection of *Legionella pneumophila*. Red, empty boxed indicate negative cultivation results. Green boxed indicate positive results including numbers of colony forming units (CFU). The number of CFUs was recalculated to their total presence per bath toy, with a detection limit of 3.3 x $10^2$ CFU/bath toy.

| A | Real bath toys | | | | | | | | | | | | | | | | | |
|---|---|---|---|---|---|---|---|---|---|---|---|---|---|---|---|---|---|---|
| *P. aeruginosa* | | $3x10^2$ | | $2x10^4$ | | $14x10^5$ | | $4x10^5$ | | $5x10^5$ | $1x10^5$ | $4x10^4$ | | $3x10^3$ | $4x10^5$ | $8x10^5$ | $3x10^3$ | |
| *Listeria* spp. | | $3x10^3$ | | | | $1x10^5$ | $2x10^4$ | | | | | | | | $4x10^2$ | $4x10^5$ | $5x10^3$ | |
| *Enterococci* spp. | | $1x10^3$ | | | | | $3x10^2$ | | | | $3x10^2$ | | | | | $1x10^5$ | | |
| Coliforms | | | | | | | | | | | | | | | | | | |
| *E. coli* | | | | | | | | | | | | | | | | | | |
| *L. pneumophila* | | $7x10^4$ | $7x10^4$ | | | | | $4x10^4$ | | | | | | $1x10^5$ | | $2x10^6$ | $7x10^3$ | |

| B | Clean water controls | | | Dirty water controls | | |
|---|---|---|---|---|---|---|
| *P. aeruginosa* | $2x10^4$ | $1x10^4$ | $1x10^3$ | $3x10^4$ | $8x10^4$ | $3x10^4$ |
| *Listeria* spp. | $8x10^3$ | $1x10^3$ | $1x10^3$ | $4x10^4$ | $6x10^4$ | $1x10^5$ |
| *Enterococci* spp. | | | | | | |
| Coliforms | $3x10^3$ | $2x10^4$ | | | | |
| *E. coli* | | | | $4x10^4$ | $1x10^5$ | $1x10^5$ |
| *L. pneumophila* | $1x10^4$ | | $7x10^3$ | $2x10^3$ | $2x10^3$ | $2x10^3$ |

**Table S7:** Carbon migration potential (MP) and biomass formation potential (BFP) of the control bath toys' plastic material. Carbon migration was measured after 1 (M1), 3 (M3), and 7 (M7) days of incubation (with water renewal on day 2, 4, 5, and 6 without measurement) and values for total organic carbon (TOC), total cell counts/cm$^2$ (TCC), as well as the proportion of assimilable organic carbon (AOC) to total organic carbon were summarized afterwards ($\sum_{M1/M3/M7}$). Standard deviations are given for experimental triplicates (*Stdev*). For the BFP, planktonic (pBFP) and biofilm (bBFP) growth after 14 d of incubation was measured.

| **Carbon migration** | **TOC [µg/cm$^2$]** | **TOC [µg/cm$^2$/day]** | **TCC [cells/cm$^2$]** | **AOC/TOC [%]** |
|---|---|---|---|---|
| $\sum_{M1/M3/M7}$ | 11.8 | - | 8.7 x 10$^7$ | 73.5 |
| *Stdev* | *0.8* | - | *1.1 x 10$^6$* | - |
| M1 | - | 5.59 | | |
| *Stdev* | - | *0.07* | | |
| M3 | - | 3.59 | | |
| *Stdev* | - | *0.62* | | |
| M7 | - | 2.59 | | |
| *Stdev* | - | *0.12* | | |
| **Biofilm formation** | **Planktonic cells/cm$^2$** | **Biofilm cells/cm$^2$** | **∑BFP [cells/cm$^2$]** | **pBFP/bBFP [%]** |
| $t_{14}$ | 3.0 x 10$^8$ | 3.6 x 10$^8$ | 6.6 x 10$^8$ | 82.5 |
| *Stdev* | *5.1 x 10$^7$* | *5.6 x 10$^7$* | *1.1 x 10$^8$* | - |

**Characteristics of tap water in the control experiment prior to and after bathing**

## Method

Water samples were taken before and after bathing in the control experiment. Total cell numbers were analyzed (FCM, SG staining; see above) and the concentrations of dissolved organic carbon (DOC), total organic carbon (TOC), total nitrogen (TN), and total phosphorus (TP) were measured. Chemical measurements were performed in a TOC-$L_{CSH}$ (SHIMADZU GmbH, Switzerland) where carbon dioxide ($CO_2$) was measured with an infrared (IR) detector after an initial catalytically burning at 720 °C. For differentiating between total and dissolved organic carbon, DOC samples were filtered (0.45 µm) before separate measurement. For the T-N measurement, $NO_x$ was measured with a chemiluminescence detector.

## Results

**Table S8: Biological and** chemical parameters for water before and after bathing. Total cell numbers per milliliter as well as concentrations for dissolved organic carbon (mg DOC/L), total organic carbon (mg TOC/L), total nitrogen (mg TN/L), and total phosphate (µg TP/L) were measured for clean water prior to bathing and dirty water after bathing including dirt and soap. Standard deviations (Stdev) are given for the measurement of three individual samples.

| | DOC [mg/L] | TOC [mg/L] | TN [mg/L] | TP [µg/L] | TCC / mL |
|---|---|---|---|---|---|
| **Before bathing** | 1.0 | 1.2 | 2.6 | 13.7 | 2.1 x $10^5$ |
| *Stdev* | *0.1* | *0.1* | *0.3* | *6.0* | *1.1 x $10^5$* |
| | | | | | |
| **After bathing** | 11.3 | 15.7 | 4.9 | 21.2 | 4.1 x $10^5$ |
| *Stdev* | *1.6* | *1.2* | *1.0* | *6.0* | *2.5 x $10^5$* |

**Table S9:** Settings for Amplicon PCR and Index PCR.

| Amplicon PCR | Temperature | Duration | Cycles |
|---|---|---|---|
| | 95°C | 5:00 min | |
| | 95°C | 0:20 min | 29 x |
| | 51°C | 0:15 min | |
| | 72°C | 0:30 min | |
| | 4°C | hold | |
| | | | |
| **Index PCR** | **Temperature** | **Duration** | **Cycles** |
| | 95°C | 3:00 min | |
| | 95°C | 0:30 min | 8 x |
| | 51°C | 0:35 min | |
| | 72°C | 0:35 min | |
| | 4°C | hold | |
